# Pancancer network analysis reveals key master regulators for cancer invasiveness

**DOI:** 10.1186/s12967-023-04435-6

**Published:** 2023-08-20

**Authors:** Mahesh Jethalia, Siddhi P. Jani, Michele Ceccarelli, Raghvendra Mall

**Affiliations:** 1 Indian Institute of Technology Kharagpur, Kharagpur, West Bengal India; 2 Centre of Brain Research, Indian Institute of Sciences, Bangalore, Karnataka India; 3 Institute of Science, Nirma University, Ahmedabad, India; 4 Department of Public Health Sciences, University of Miami, Miami, FL USA; 5 Sylvester Comprehensive Cancer Center, University of Miami, Miami, FL USA; 6 St. Jude Children’s Hospital, Memphis, TN USA; 7 Biotechnology Research Center, Technology Innovation Institute, P.O. Box 9639, Abu Dhabi, United Arab Emirates

**Keywords:** Cancer systems biology, Invasiveness, Master regulators, Signaling pathways, Consensus clustering, ARACNE, RGBM, VIPER

## Abstract

**Background:**

Tumor invasiveness reflects numerous biological changes, including tumorigenesis, progression, and metastasis. To decipher the role of transcriptional regulators (TR) involved in tumor invasiveness, we performed a systematic network-based pan-cancer assessment of master regulators of cancer invasiveness.

**Materials and methods:**

We stratified patients in The Cancer Genome Atlas (TCGA) into invasiveness high (INV-H) and low (INV-L) groups using consensus clustering based on an established robust 24-gene signature to determine the prognostic association of invasiveness with overall survival (OS) across 32 different cancers. We devise a network-based protocol to identify TRs as master regulators (MRs) unique to INV-H and INV-L phenotypes. We validated the activity of MRs coherently associated with INV-H phenotype and worse OS across cancers in TCGA on a series of additional datasets in the Prediction of Clinical Outcomes from the Genomic Profiles (PRECOG) repository.

**Results:**

Based on the 24-gene signature, we defined the invasiveness score for each patient sample and stratified patients into INV-H and INV-L clusters. We observed that invasiveness was associated with worse survival outcomes in almost all cancers and had a significant association with OS in ten out of 32 cancers. Our network-based framework identified common invasiveness-associated MRs specific to INV-H and INV-L groups across the ten prognostic cancers, including COL1A1, which is also part of the 24-gene signature, thus acting as a positive control. Downstream pathway analysis of MRs specific to INV-H phenotype resulted in the identification of several enriched pathways, including Epithelial into Mesenchymal Transition, TGF-β signaling pathway, regulation of Toll-like receptors, cytokines, and inflammatory response, and selective expression of chemokine receptors during T-cell polarization. Most of these pathways have connotations of inflammatory immune response and feasibility for metastasis.

**Conclusion:**

Our pan-cancer study provides a comprehensive master regulator analysis of tumor invasiveness and can suggest more precise therapeutic strategies by targeting the identified MRs and downstream enriched pathways for patients across multiple cancers.

**Graphical Abstract:**

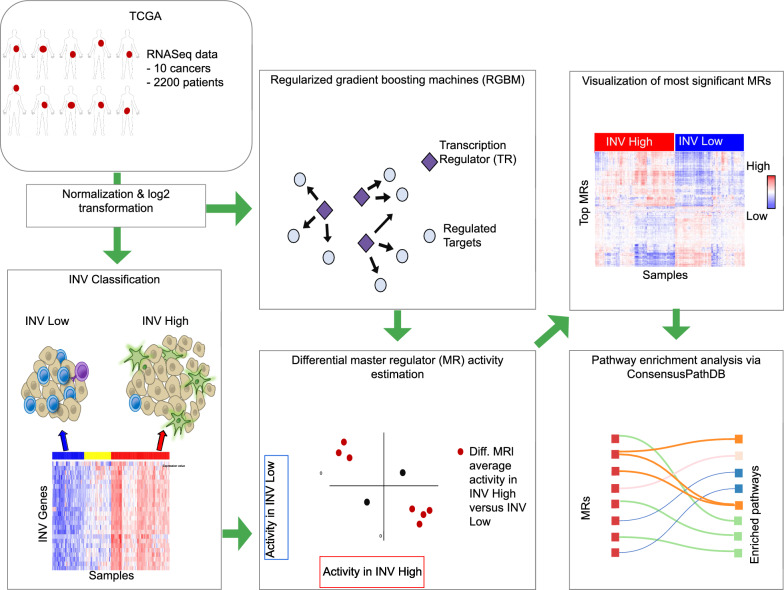

**Supplementary Information:**

The online version contains supplementary material available at 10.1186/s12967-023-04435-6.

## Introduction

Cancer is one of the leading causes of death worldwide, accounting for over 10 million deaths annually [1]. Once cells are damaged then evading programmed cell death [2], invading local tissue (activating invasion) and metastasizing are hallmarks of cancer [3]. Cancer invasiveness is a phenotype usually associated with a worse survival prognosis [4]. In this context, several invasiveness-associated gene signatures have been reported [5–7] for individual cancer types. However, in [4], the authors devised a robust 24-gene signature through comprehensive pan-cancer analysis. This gene signature includes *COL11A1, POSTN, EPYC, ASPN, COL10A1, THBS2, FAP, LOX, SFRP4, INHBA, MFAP5, GREM1, COMP, VCAN, COL5A2, COL5A1, TIMP3, GAS1, TNFAIP6, ADAM12, FBN1, SULF1, COL1A1* and *DCN.* While a pan-cancer analysis of invasiveness-associated dysregulated molecular features, including genomic, epigenomic, transcriptomic, proteomic, and metabolomic features, has been conducted in [4], the clinical impact of invasiveness for patient stratification and the mechanisms governing the transcriptional regulations and their associated pathway alterations are still poorly understood. Thus, it is imperative to determine the pan-cancer prognostic relevance of invasiveness and identify key driver genes and their associated downstream mechanisms to design better therapeutic strategies.

In this study, we developed a systematic framework using 32 tumor lineages from The Cancer Genome Atlas (TCGA) [8] to characterize the prognostic implications of invasiveness. Using the 24-gene signature and a consensus clustering approach [9–15], we classified the tumor samples into Invasiveness High (INV-H), Invasiveness Medium (INV-M), and Invasiveness Low (INV-L) groups for each cancer type to determine the prognostic association i.e. correlation between INV-H and INV-L clusters and overall survival (OS). Our working hypothesis is to determine whether the intrinsic activation of transcriptional regulators (TRs) is involved in sustaining the oncogenic process influencing invasiveness.

A necessary condition for tumor progression, metastasis, and drug resistance is transcriptional dysregulation [16, 17]. A majority of the cancer driver genes are TRs [18]. TRs are largely dysregulated due to genomic alterations in their regulatory proteins, which in turn can modulate the expression of their target genes, referred to as their ‘regulon.’ TRs identified as key oncogenic drivers whose activity patterns are influential to a patient’s clinical diagnosis [19] are referred to as master regulators (MRs). In the recent literature, there are methods, such as Netfactor [20] and [16], that take a consensus-based approach to identify signature-specific MRs. In this work, to be comprehensive, we use a consensus of four different network-based master regulator analysis (MRA) pipelines [21–31] on publicly available RNA-Seq data from TCGA. We identified MRs specific to INV-H and INV-L phenotypes with similar activity patterns across multiple cancers where invasiveness has prognostic relevance. Extensive validation of activities of MRs was done on sets from two different sources, i.e., the not significantly prognostic cancer types from TCGA and the datasets from the Prediction of Clinical Outcomes from Genomic Profiles (PRECOG) repository [32]. Finally, we perform downstream analysis of the MRs specific to INV-H (associated with worse OS) using ConsensusPathDB [33] to discover enriched pathways, several of which are potential candidates for targeted therapy.

## Materials and methods

### Data acquisition and normalization

RNA-Seq data from the TCGA website (https://www.cancer.gov/tcga) were downloaded for each cancer c separately using TCGAbiolinks (v2.22.3) through the ‘GDCqeury()’ function. This resulted in the STAR-protocol [10] based raw count matrix. For each c, the patient samples (count matrix) were quantile normalized using preprocessCore (v1.56.0) R package and then log2 transformed (see Additional file 1: Figure S2). The processed RNA-Seq data for each cancer was represented as ${D}^{c}=\left[{g}_{1}^{c},{g}_{2}^{c},\dots ,{g}_{p}^{c}\right]$, where ${D}^{c}$ represents the RNA-seq matrix, ${g}_{i}^{c}$ represents the ith gene’s expression profile as a column vector and has dimension ${N}_{c}\times 1$. Here ${N}_{c}$ corresponds to the number of patient samples for a particular cancer c. The dimension of the RNA-seq matrix is ${N}_{c}\times p$, where p is the total number of genes in the expression matrix. The RNA-seq data from 32 primary solid tumors (TP) consisting of over 9000 samples in total were used in our analysis. Owing to the lack of TP samples in SKCM, we included the metastatic samples (TM) in the SKCM dataset. Gene symbols were converted to official HUGO Gene Nomenclature Committee gene symbols, and genes without gene symbols or gene information were excluded. This resulted in p = 23,216 genes, including TRs for each cancer c.

### Validation datasets

For the out-of-box validation of the activation profiles of the Master Regulators (MRs), we accumulated independent test sets from the PRECOG repository. We selected eight datasets, each corresponding to a different cancer type and the largest available dataset for a particular cancer type as the validation set. These included GEO Accession Id: GSE32894 [34] for Bladder Urothelial Carcinoma (BLCA), GSE3494 [35] for Breast Invasive Carcinoma (BRCA), GSE39582 [36] for Colon adenocarcinoma (COAD), GSE108474 [37] for Glioblastoma multiforme (GBM), GSE65858 [38] for Head and Neck squamous cell carcinoma (HNSC), GSE72094 [39] for Lung adenocarcinoma (LUAD), GSE9891 [40] for Ovarian serous cystadenocarcinoma (OV) and GSE65904 [41] for Skin Cutaneous Melanoma (SKCM). These eight datasets consisted of 224, 251, 579, 490, 270, 398, 278, and 210 tumor samples with 21817, 19980, 23216, 23216, 23216, 20061, 23216, and 23216 genes, respectively, and were normalized using quantile normalization [36], followed by log2 transformations. These normalized datasets and INV clusters for each sample within each cancer c were estimated using the 24-gene signature as discussed in the following subsection.

### Cancer invasiveness clusters

**Fig. 1:**
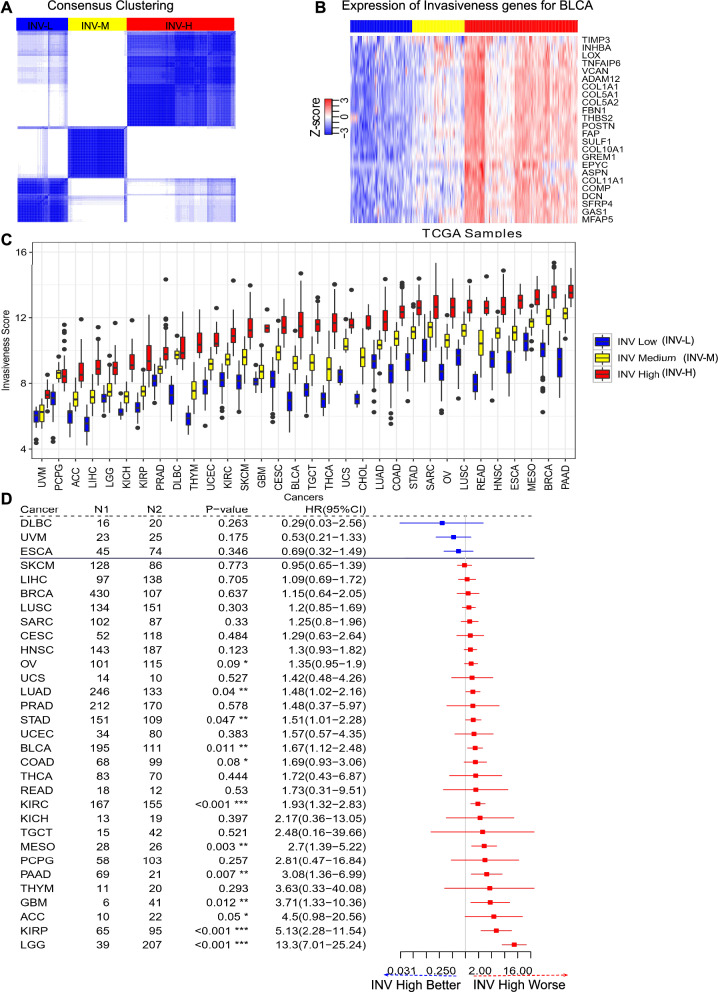
**A** Consensus Clustering based on Invasiveness gene set. The ‘red’ corresponds to INV High, ‘yellow’ to INV Medium and ‘blue’ to INV Low clusters. **B** Expression of the 24 invasiveness genes in BLCA cancer. The ‘red’ corresponds to INV High, ‘yellow’ to INV Low and ‘blue’ to INV Low groups. **C** Invasiveness score distribution in INV High, Low and Medium groups identified as consensus clusters. **D** Differential association of Invasiveness phenotype (INV High vs INV Low) with survival prognosis in TCGA patients. Here **N1** and **N2** represent the number of samples in INV Low and High clusters respectively, **HR** corresponds to hazards ratio and **CI** represents confidence intervals. The ‘*’ corresponds to significance of association as obtained from the univariate survival analysis model. ‘*’ → 0.01 < P-value < 0.1, ‘**’ → 0.001 < P-value < 0.01 and ‘***’ → P-value < 0.001

An unsupervised consensus clustering based on a robust gene set of 24 invasiveness-relevant genes was performed for each cancer type separately using the ConsensusClusterPlus (v1.58.0) R package with the following parameters: 5000 repeats, a maximum of six clusters and agglomerative hierarchical clustering with the distance method set as Ward (‘ward.D2’) distance. This method has previously successfully identified optimal prognostic clusters for the pancancer immunologic constant of rejection [42–45] and pancancer panoptosis phenotype [2]. The optimal number of clusters () for the best segregation of samples based on the invasiveness signature was initially determined heuristically using the Calinski-Harbasz criterion [46]. With the intent to compare cancer samples with a highly active invasiveness phenotype with those that have a relatively inactive invasiveness phenotype, the cluster with the highest average expression of invasiveness gene signature was designated as ‘Invasiveness high’ (INV-H), while the cluster with the lowest average expression of invasiveness gene signature was designated ‘Invasiveness low’ (INV-L). All samples in the intermediate cluster(s) were defined as an ‘Invasiveness medium’ (INV-M, see Fig. 1B). Tumor samples were annotated with an invasiveness score, defined as the average expression of the 24-gene signature panel in a particular tumor sample and mathematically depicted as:

1 $${score}_{i}^{c}=\frac{1}{24}{\sum }_{j=1}^{24}{g}_{j,i}^{c}$$

### Survival analysis

Overall survival (OS) from the TCGA clinical data resource was used to estimate the hazard ratios for survival analysis. For each cancer c, patients with less than one day of follow-up were removed, and the survival data were censored after a follow-up duration of ten years. The hazard ratios (HR) between INV-H and INV-L clusters, their corresponding confidence intervals, and P-values were estimated using a univariate survival analysis model for each cancer *c* using the ‘analyze_survival’ function from survivalAnalysis (v0.2.0) R package [47]. We used the ‘kaplan_meier_plot’ function to visualize the Kaplan–Meier plots for cancers with significant prognosis (see Additional file 1: Figure S1). A forest plot was generated using the forestplot (v2.0.1) R package (see Fig. 1D). The cancer type cholangiocarcinoma (CHOL, no death event in INV-L) was excluded before the generation of the forest plot, as the number of deaths in the two comparison groups (INV-H versus INV-L) was too small for survival estimation. Cancers with a P-value < 0.1 and a total number of tumor samples > 50 in INV-H plus INV-L groups were identified as cancers where invasiveness had a significant prognostic value.

Figure 1 depicts a sample consensus clustering for BLCA (Bladder Urothelial Carcinoma), the gene expression profile of the 24-gene signature across the tumor samples from BLCA, the variations in invasiveness score between the invasiveness clusters across 32 different cancers, and a forest plot highlighting the pancancer prognostic relevance of invasiveness phenotype.

### Master regulator analysis pipeline

**Fig. 2:**
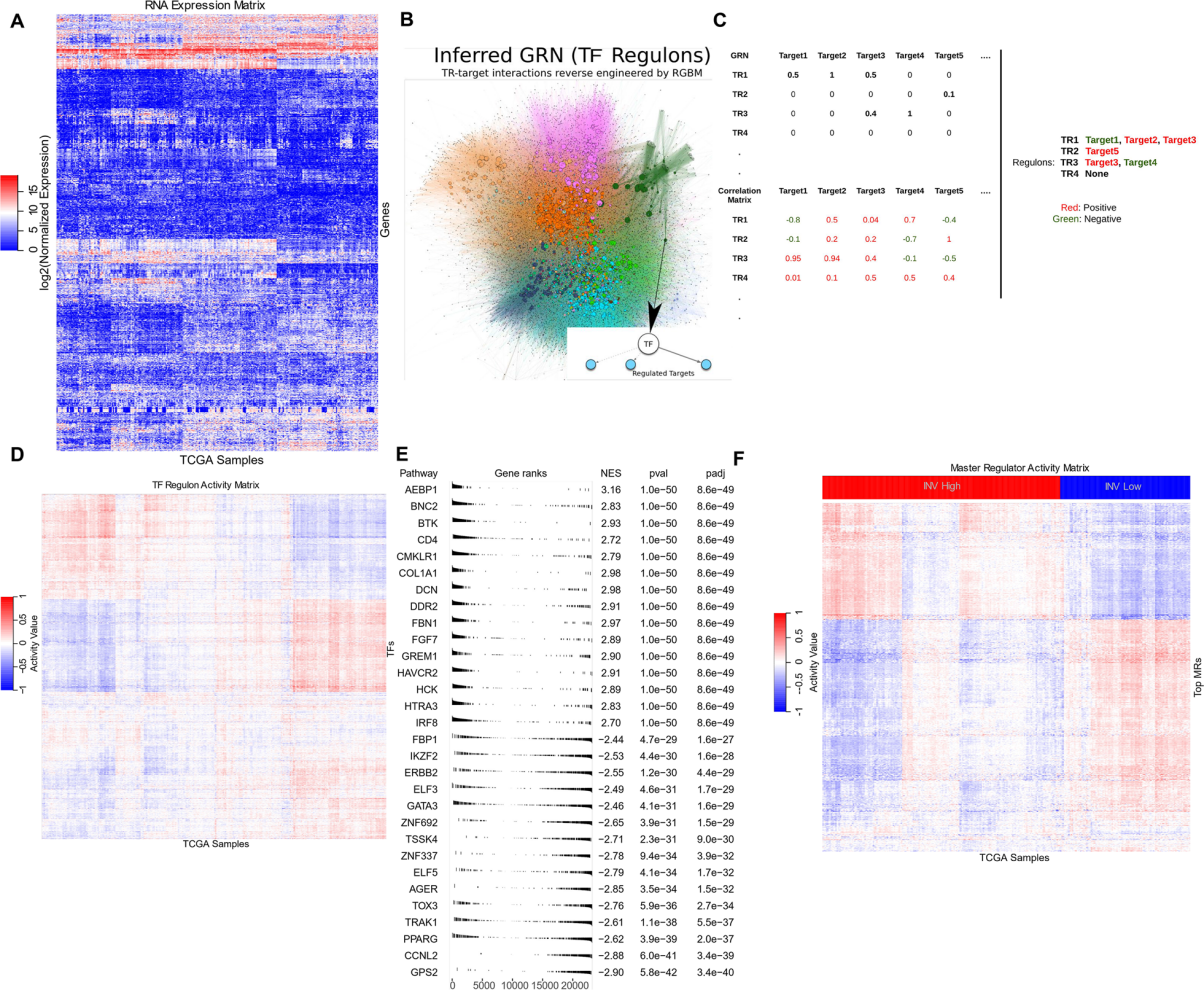
Master Regulator Analysis Pipeline for a pancancer invasiveness phenotype. **A** A sample RNA-Seq matrix of genes vs samples where rows represents genes and columns represent tumor samples. **B** Reverse-engineered gene regulatory network using RGBM technique. Each big blog represents a transcription factor (TFs) and the small dots represent target genes. The regulatory network is divided into communities (color-coded) using Louvain clustering algorithm. **C** An example of the adjacency list corresponding to the inferred GRN as well as the correlation matrix between the TFs and target genes based on the RNA-seq matrix. **D** The sample-TF activity matrix as estimated by **Eq. **2 where rows are TFs and columns are tumor samples. **E** Significantly enriched TRs (FDR-adjusted P-value < 0.05) referred to as master regulators (MRs) identified by using the FGSEA method. **F** The sample-MR activity matrix extracted from sample-TF activity matrix. The activity matrix has block diagonal structure with some MRs having high activity in INV-H but low activity in INV-L while other MRs vice versa activity profiles

There have been several methods in the literature [48, 49] to perform MR analysis (MRA). The primary component for MRA is to infer a ‘high-quality’ gene regulatory network (GRN) consisting of TR-target gene interactions (regulons) from RNA-Seq data (see Fig. 2A, B). This is one of the central problems in computational biology, and several techniques have been proposed, including the mutual information-based method ARACNE [21] and tree-based machine learning techniques such as GENIE [22] and regularized gradient boosting machine (RGBM) [24]. ARACNE [21] formulates the problem of inferring GRN using an information-theoretic approach, including a combination of mutual information and data-processing inequality steps to identify possible TF-target interactions from RNA-seq data and then filter out spurious TR-target connections. However, both GENIE and RGBM formulate the GRN reconstruction task as a regression task: F(X_TR_) = Y_t_, where X_TR_ represents the matrix of gene expression of transcriptional regulators and Y_t_ corresponds to the expression profile of a given target (t) gene. Both GENIE and RGBM use a tree-based machine learning framework to solve the underlying regression task. While GENIE [22] uses a random-forest approach, RGBM [24] utilizes the gradient boosting machine In [27], through an open-science competition (DREAM Challenge), the authors compared various GRN inference methods on several synthetic and real datasets. In [24], the authors illustrated the superior performance of RGBM for the DREAM Challenge networks over other tree-based methods such as GENIE. Hence, RGBM is the primary GRN inference technique used in the present work.

Another key ingredient of the MRA pipeline is to estimate enrichment/activity scores for TRs in a given tumor sample, taking into consideration its regulon (see Fig. 2C). This is essential to identify significantly differentially enriched/activated TRs (referred to as MRs, see Fig. 2D and F, respectively). RGBM utilize a simplistic difference in the average expression of positively and negatively regulated targets to estimate the activity of a TR as defined below:

2 $${Act\left(TR,i\right)}^{C}=\frac{1}{U}{\sum }_{k=1}^{U}{t}_{ki}^{p}-\frac{1}{V}{\sum }_{j=1}^{V}{t}_{ji}^{n}$$

Here ${t}_{ki}^{p}$ is the expression level of the *k*th positive target of a TR in the *i*th sample, ${t}_{ji}^{n}$ is the expression level of the *j*th negative target of the TR in the *i*th sample, *U* (*V*) is the number of positive (negative) targets present in the regulon of the considered TR. If *Act(TR,i)* > 0, the TR is *active* in that particular sample. If *Act(TR,i)* < 0, then the TR is inversely activated and if *Act*(*TR,i*) ≈ 0 it is not considered active. In contrast, methods such as virtual inference of protein activity by enriched regulon analysis (VIPER) [50] and MARINA [51] utilize a dedicated algorithm formulated to estimate TR activity taking into account the TR mode of action, the TR-target gene interaction confidence and the pleiotropic nature of each target gene regulation. Moreover, there exist single sample gene set enrichment analysis [29] techniques such as gene set variation analysis (GSVA [31]) and fast gene set enrichment analysis (FGSEA [52]) to estimate the enrichment score for each TR in a given sample.

Recently, techniques such as Netfactor [20] have been devised which take a consensus-based approach to identifying signature-specific MRs. This is because it was shown in [16] that TR regulons estimated by a consensus approach are more robust for downstream tasks with less risk of being influenced by false positives. Following the same notions, we determined MRs specific to INV-H and INV-L phenotypes by taking a consensus (in this case an intersection) of the MRs identified by four different MRA techniques: (a) RGBM + FGSEA, (b) RGBM + GSVA, (c) RGBM + VIPER and (d) ARACNE + VIPER.

Thus, in our pipeline, we use two different class of GRN inference techniques, one based on information theory (ARACNE) and other based on gradient boosting machine (RGBM),and three different gene set enrichment/activity estimation techniques to identify the key MRs specific to INV High and INV Low for each c.

Figure 2 provides an example of an RGBM + FGSEA-based MRA pipeline.

### Transcriptional regulators

We wanted to select TRs from an expanded pool of candidates, including genes involved in modulating the rate, frequency, and extent of cellular DNA-templated transcription. Thus, we selected all genes annotated with the GO:0006355 (regulation of transcription) [53] gene ontology term. We had 3,674 TRs, including transcription factors (TFs), receptors, growth factors, kinases, signal transduction proteins, transcription co-activators, and cofactors. Previous works using hubs of networks were focused on either surface receptors i.e. the receptors interactome to identify active ligand-receptor pairs [54], or signaling molecules, including Sigmaps [55] and not just TFs.

### Inferring gene regulatory networks

Given ${D}^{c}$, we inferred GRN between the TRs and the target genes (i.e. TR-target edges, see Fig. 2B) using two different class of techniques, namely RGBM [24] and ARACNE [21]. The inferred GRNs were unsigned and weighted. RGBM belongs to the class of machine learning techniques based on feature selection where the expression vector of each target gene (i.e., t) is considered as a dependent variable (${Y}_{t}={g}_{t}^{c}$) and the expression matrix corresponding to the list of TRs are the independent variables (X_TR_). The goal of RGBM is to detect linear/non-linear TR-target interactions using a gradient boosting procedure [56] with a decision tree [57] as a base learner. ARACNE, on the other hand, is based on concepts of mutual information (MI(g^c^_TF_, g^c^_t_)) and prevents indirect transitive interactions using an information-theoretic property, the data processing inequality [21]. Using a bootstrapping procedure, ARACNE can also provide the strength (in terms of statistical significance) of a TR-target interaction. For quality control, we remove those TRs whose regulon size is less than 10 in both RGBM and ARACNE inferred GRNs. We used the RGBM (v1.0.10) and corto (v1.1.11) packages in R to implement RGBM and ARACNE methods for GRN inference respectively.

### Scoring TR activities

Given ${D}^{c}$ and the GRN (${G}^{c}$) for a particular cancer c, the level of activity of a TR in a sample can be estimated as a function of the collective mRNA levels of its targets as illustrated in RGBM (as illustrated in Eq. 2) and VIPER [50]. In RGBM, the regulon of a TR (see Fig. 2B) was divided into positively regulated targets and negatively regulated targets by performing a Pearson correlation between the expression of the TR (g^c^_TR_) and the expression of the target genes (g^c^_t_) in its regulon across all the samples for that cancer c (see Fig. 2C). The targets with positive correlations were considered as activated targets and the targets with negative correlations were identified as repressed targets in the TR’s regulon. This simplistic formulation for TR activity calculation was shown to be effective for the identification of differentially active TRs (i.e., MRs) [24, 58] (as illustrated in Eq. 2) and VIPER [50]. In RGBM, the regulon of a TR (see Fig. 2B) was divided into positively regulated targets and negatively regulated targets by performing a Pearson correlation between the expression of the TR (g^c^_TR_) and the expression of the target genes (g^c^_t_) in its regulon across all the samples for that cancer c (see Fig. 2C). The targets with positive correlations were considered as activated targets and the targets with negative correlations were identified as repressed targets in the TR’s regulon. This simplistic formulation for TR activity calculation was shown to be effective for the identification of differentially active TRs (i.e., MRs) [58].

### Gene-set enrichment analysis and MR selection

In VIPER [50], a probabilistic framework that directly integrates the target mode of regulation, i.e., whether targets are activated or repressed, confidence in regulator-target interactions and target overlap between different regulators, is utilized to compute the enrichment of a TRs’ regulon. A normalized enrichment score (NES) is computed analytically, assuming that in the null situation, the target genes are uniformly distributed on the gene expression signature. Since there is extensive co-regulation of gene expression taking place in the cell, this assumption has been demonstrated to never hold [50], and this is the reason why a null model based on sample permutations is used. To generate NES for TRs in INV-H, we use the INV-M samples as a set of reference samples. The corresponding null model based on sample permutations can be obtained with the function ‘viperSignature’ function in the viper (v1.32.0) R package. Similarly, to generate the NES for TRs in INV-L, we again use the INV-M samples as a set of reference samples. Since VIPER expresses activity for all the TRs in the same scale, i.e., NES, we can now perform differential analysis using a Bayesian statistical framework such as LIMMA [59] package (v3.54.1) in R to identify differentially activated TRs (MRs) between INV-H and INV-L samples for a particular c.

In FGSEA [52], to identify the differentially active TR regulons between INV-H and INV-L primary tumor samples, we first estimate the average mRNA level difference of each gene between the two groups. This difference represents the fold change score (FC-score). All the genes are then sorted in decreasing order based on the estimated FC-score. To determine the enrichment score for specific TR regulons, we then use the ‘fgsea’ function in the fgsea (v1.24.0) package in R [52]. It implements an algorithm to calculate the empirical NES null distributions simultaneously for all the gene-set sizes (TR regulons), which allows up to several hundred times faster execution time compared to the original GSEA [29] implementation. This also enables FGSEA to provide statistical significance associated with the NES scores for TRs. We select TRs with FDR-adjusted [60] P-values ≤ 0.05 and |NES^c^|> 1 for all cancer types as the statistically significant differentially enriched TR regulons i.e. differentially activated MRs (see Fig. 2E). Here |NES^c^| is used for absolute values of the NES score for a cancer c. Figure 2F highlights the activity of the MRs indicating there are some MRs with high activity in the INV-H samples but low activity in the INV-L samples and vice-versa.

In GSVA [31], a non-parametric, unsupervised technique is used to estimate TR regulon enrichment scores as a function of genes inside and outside the regulons, analogously to a competitive gene set test. We use the ‘gsva’ function in the GSVA (v1.46.0) package in R providing the expression information, TR regulons, a maximum and minimum size of a regulon as input, and keeping all other parameters to default settings. We obtain a sample-specific enrichment score for each TR regulon, which can now be utilized to perform differential analysis using a Bayesian statistical framework such as LIMMA to determine the differentially activated TRs (MRs) between INV-H and INV-L samples for a cancer type c.

### Pathway and GO term enrichment analysis

We use ConsensusPathDB for the functional (GO Term) and pathway enrichment analysis of MRs across the prognostic cancer types for INV-H and INV-L phenotype separately (latest version [33]). ConsensusPathDB allows us to perform over-expression analysis on top of differentially activated MRs to identify significantly enriched molecular functions (m), cellular components (c), biological processes (b), and pathways (p). The advantage of using ConsensusPathDB over a popular tool like DAVID [61] is that it provides the option to search through multiple databases (different types of interactions) to find enriched pathways unlike DAVID, which only uses the KEGG database. Moreover, unlike Ingenuity Pathway Analysis, ConsensusPathDB is open-source software available for such enrichment analysis. Since we consider well-annotated TRs, we only include databases such as WikiPathways, Reactome, and KEGG, all available in ConsensusPathdb, for downstream enrichment analysis. Visualizing the enriched pathways obtained via ConsensusPathDB is performed using the func2vis package (v1.0.2) in R [44].

The list of all abbreviations used in the manuscript and the full names of all the cancer types from TCGA are available in Tables 1 and 2 respectively.

**Table 1:** List of notations and abbreviations used

TR	Transcription regulator
MR	Master Regulator
GRN	Gene Regulatory Network
INV	Immunologic Constant of Rejection
NES	Normalized Enrichment Score
MRA	Master Regulator Analysis
TCGA	The Cancer of Genome Atlas
PRECOG	Prediction of Clinical Outcomes for Genomics profiles
RGBM	Regularized Gradient Boosting Machines
GSEA	Gene Set Enrichment Analysis
FGSEA	Fast Gene Set Enrichment Analysis
GSVA	Gene Set Variation Analysis
VIPER	Virtual inference of protein activity by enriched regulons
INV-H	Highest expression of INV genes
INV-L	Lowest expression of INV genes
INV-M	Medium expression of INV genes

**Table 2:** TCGA cancer abbreviations

Cancer	Cancer type	Full name
LGG	Primary	Brain low grade glioma
KIRC	Primary	Kidney renal cell carcinoma
SKCM	Metastatic	Skin cutaneous melanoma
STAD	Primary	Stomach adenocarcinoma
COAD	Primary	Colon adenocarcinoma
ACC	Primary	Adrenocortical carcinoma
BLCA	Primary	Bladder urothelial cancer
BRCA	Primary	Breast invasive carcinoma
CESC	Primary	Cervical squamous cell carcinoma and endocervical adenocarcinoma
CHOL	Primary	Cholangiocarcinoma
ESCA	Primary	Esophageal carcinoma
GBM	Primary	Glioblastoma multiforme
HNSC	Primary	Head and neck squamous cell carcinoma
KICH	Primary	Kidney chromophobe
PAAD	Primary	Pancreatic adenocarcinoma
THYM	Primary	Thymoma
KIRP	Primary	Kidney renal papillary cell carcinoma
LIHC	Primary	Liver hepatocellular carcinoma
LUAD	Primary	Lung adenocarcinoma
DLBC	Primary	Lymphoid neoplasm diffuse large b-cell lymphoma
MESO	Primary	Mesothelioma
OV	Primary	Ovarian cystadenocarcinoma
PCPG	Primary	Pheochromocytoma and paraganglioma
PRAD	Primary	Prostate adenocarcinoma
READ	Primary	Rectum adenocarcinoma
TGCT	Primary	Testicular germ cell tumors
THCA	Primary	Thyroid carcinoma
UCS	Primary	Uterine carcinosarcoma
UVM	Primary	Uveal melanoma
UCEC	Primary	Uterine corpus endometrial carcinoma
LUSC	Primary	Lung squamous cell carcinoma
SARC	Primary	Sarcoma

### Multi-cancer master regulator activity matrix

Once we have identified the MRs specific to INV-H and INV-L groups based on the cancers where invasiveness phenotype is prognostically relevant, we illustrate that activity patterns of these MRs in invasiveness neutral cancers (INV-N) i.e. cancers where invasiveness phenotype is not significantly associated with overall survival. We estimate the MR activity in each cancer sample for a particular cancer c using the RGBM + FGSEA approach. We consider the activities of all these MRs as a column vector grouped by the activity of MRs specific to INV-L first and followed by the activities of MRs specific to INV-H (see Fig. 5A). We collect all the column vectors of MR activity in the INV-L patient samples for the 22 different INV-N cancers and concatenate them to create the INV-L activity matrix. We perform hierarchical clustering across the column vectors with the distance method set as Ward (‘ward.D2’) distance. Similarly, we collect all the column vectors of MR activity in the INV-H patient samples for the 22 different INV-N cancers and concatenate them to create the INV-H activity matrix. We again perform hierarchical clustering across the column vectors. Once we have the hierarchically clustered INV-L and INV-H matrices for the INV-N cancers, we concatenate (along the column axis) the two to obtain the multi-cancer MR activity matrix (see Fig. 5A) for INV-N cancers.

We use the same procedure to create the multi-cancer MR activity matrix (see Fig. 5B) for the PRECOG validation cancer datasets.

## Experimental results

### Prognostic impact of invasiveness clusters in different cancers subtypes

To improve our understanding of the role of invasiveness in cancer and to determine whether invasiveness has prognostic value in this context, we evaluated the 24-gene invasiveness signature across 32 cancer types from TCGA. To group tumor samples based on the gene expression profiles of the invasiveness markers, we performed unsupervised consensus clustering for each c separately (BLCA provided as an example; see Fig. 1A). The consensus clustering identified three clusters referred to as INV-H, INV-M, and INV-L, where tumors belonging to the INV-H cluster had a majority of invasiveness markers highly expressed, thereby suggesting the possibility of enhanced invasiveness, and vice versa for the INV-L cluster (see Fig. 1B).

We also estimated a score referred to as the invasiveness score for each tumor sample. The invasiveness score was quantified as the average expression of the 24-gene signature in tumor samples (see Eq. 1). We observed that the invasiveness score varied among the tumor samples for a particular c, reflective of the intratumor heterogeneity (see Fig. 1C). The difference between the highest and lowest invasiveness varied between the different cancer types. We noticed a stark contrast between the median invasiveness scores in INV-H and INV-L groups for cancers such as BLCA, COAD, PAAD, OV, etc. (see Fig. 1C). We, therefore, sought to investigate the clinically relevant question i.e. how the presence of two contrasting invasiveness clusters (INV-H vs INV-L) contributed to the survival and how it varied across multiple cancer types.

To determine the clinical relevance of invasiveness clusters, we performed a univariate survival analysis for each of the 32 different cancers comparing the survival of patients in the INV-H cluster (treatment group) to that of patients in the INV-L cluster (control group). The quantitative difference in survival was measured via hazard ratio (HR) along with a 95% confidence interval (denoted in parentheses, see Fig. 1D). An HR above a value of 1 suggested that patients with tumors in the INV-H cluster had worse survival than patients in the INV-L cluster; an HR below a value of 1 suggested that patients with tumors in the INV-H cluster had better survival prognosis than patients in the INV-L cluster.

The invasiveness high phenotype was predominantly associated with worse OS across the majority of cancers. However, there were ten cancer types for which INV-H was significantly prognostic, including LGG (P-value <  < 0.001), KIRP (P-value <  < 0.001), PAAD (P-value = 0.007), MESO (P-value = 0.003), KIRC (P-value <  < 0.001), COAD (P-value = 0.08), BLCA (P-value = 0.011), STAD (P-value = 0.047), LUAD (P-value = 0.04) and OV (P-value = 0.09) with HR of 13.3 (7.01–25.24), 5.13 (2.28–11.54), 3.08 (1.36–6.99), 2.7 (1.39–5.22), 1.93 (1.32–2.83), 1.69 (0.93–3.06), 1.67 (1.12–2.48), 1.51 (1.01–2.28), 1.48 (1.02–2.16), 1.35 (0.95–1.9) respectively as observed from the forest plot in Fig. 1D and Kaplan–Meier plot in Additional file 1: Figure S1. We also observed a significant prognostic association for ACC (P-value = 0.05) and GBM (P-value = 0.012). Still, since the total number of samples in the INV-H (N1) and INV-L (N2) was < 50 samples, we did not consider these cancers further in our analysis.

Together these results suggested that patients could be clustered into three different groups for each c: INV-H, INV-M, and INV-L w.r.t gene expression profiles of invasiveness markers. Moreover, INV-H and INV-L clusters were associated with OS for 10 different cancer subtypes, highlighting their clinical relevance.

### GRN comparison and consensus MRs

A detailed comparison of the inferred GRNs of RGBM and ARACNE methods (per cancer c) is available in [43]. It was observed in [43] that for each c with a large number of RNA-seq samples, the RGBM and ARACNE inferred GRNs tend to have a higher Jaccard coefficient. Jaccard coefficient is a measure of similarity, taking values between [0,1] and higher coefficient suggested higher similarity of the GRNs owing to similar sets of edges.

In this work, we used four different pipelines for performing MRA: (a) RGBM + FGSEA; (b) RGBM + GSVA; (c) RGBM + VIPER, and (d); ARACNE + VIPER and took a consensus, i.e., intersection of the MRs determined by these varied pipelines as the differentially activated MRs between INV-H and INV-L samples for a particular c. For the RGBM + FGSEA method, we used the |NES^c^|> 1.0 and FDR-adjusted p-value ≤ 0.05 as the selection criterion for identifying the differentially activated TRs (MRs). Moreover, in RGBM + FGSEA method, the activity scores for all the TR regulons were normalized in the range [-1, 1] by dividing the positive activity values by maximum positive activity and negative activity values with the absolute minimum of negative activity (for each c, see Fig. 2D, Eq. 2). The raw activity profiles of TR regulons for each c follow a normal distribution as observed in Additional file 1: Figure S3. However, for the other 3 pipelines to be less restrictive, we selected all TRs with FDR-adjusted P-value ≤ 0.05 when comparing the enrichment scores between INV-H and INV-L samples as our MRs.

We obtained a total of 737, 590, 547, 279, 741, 829, 744, 661, 537, and 413 consensus MRs for LGG, KIRP, PAAD, MESO, KIRC, COAD, BLCA, STAD, LUAD and OV respectively by taking an intersection of the MRs identified by the 4 different MRA pipelines. Henceforth, we use terms such as consensus MRs or common MRs, or MRs interchangeably for differentially activated TRs common to the 4 MRA pipeline in the rest of the manuscript.

### MR activities across primary tumors for invasiveness phenotype

**Fig. 3:**
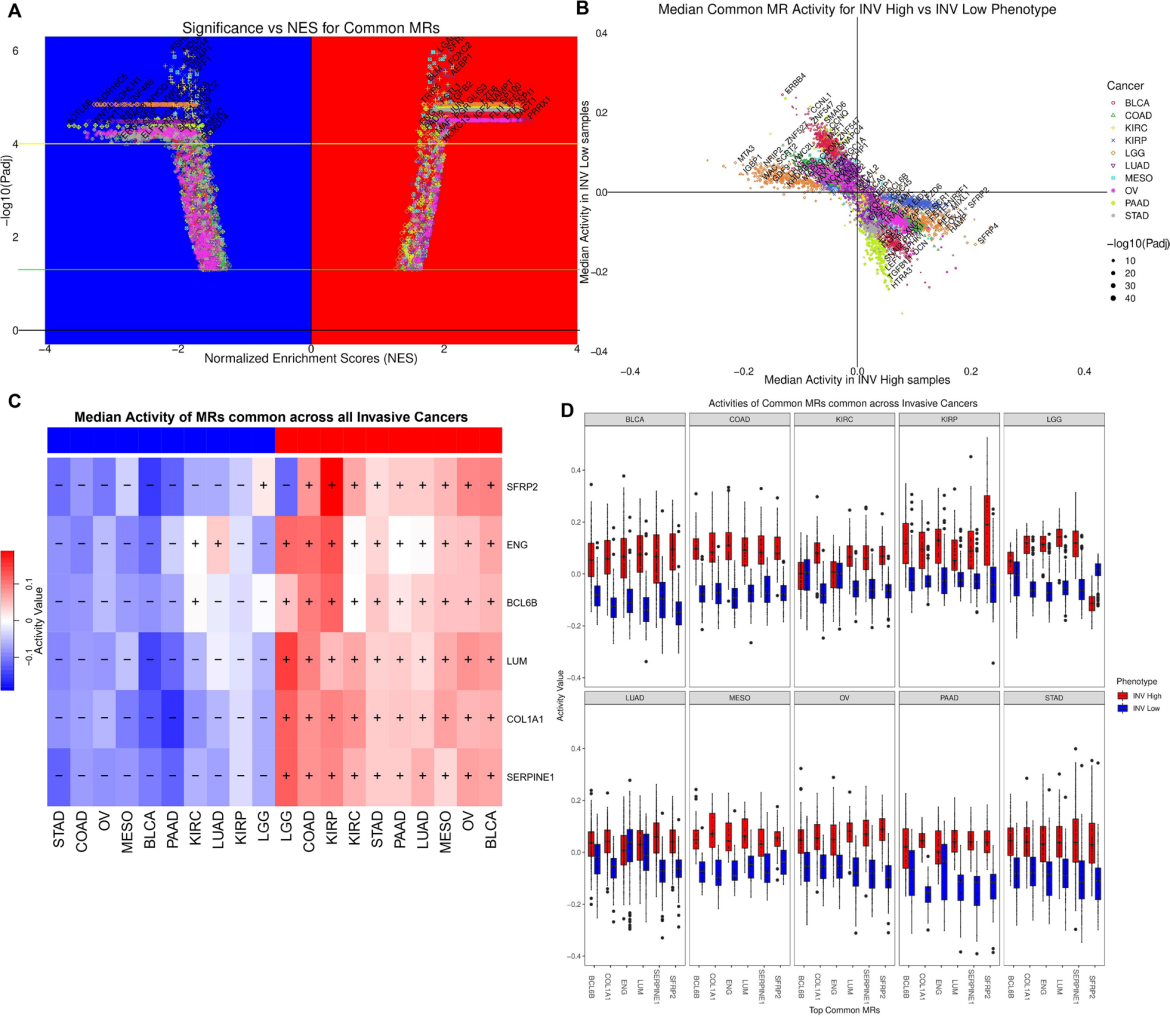
Top MRs common across all the 10 cancers where invasiveness has a significant prognostic impact. **A** Volcano plot highlighted the NES for TRs along the x-axis and significance of enrichment along the y-axis across the 10 cancers of interest. The TRs above the ‘yellow’ line are considered as MRs. **B** Median activity of MRs in the INV High and INV Low samples for different cancers. The size of the plot reflects the significance of enrichment. **C** The six MRs common across all the ten prognostic cancers along with their median activity in INV High and INV Low samples. **D** The variation in activities of the six MRs common across all the ten cancers depicted as boxplots

We highlight the NES scores for common MRs, as determined by the FGSEA method, for each cancer c as a volcano plot in Fig. 3A. We demonstrated the median activity across INV-H and INV-L samples of these MRs for each cancer c in Fig. 3B. Moreover, an MR does not have to be a TR in all the 10 cancers to be considered in our analysis. We observed that MRs whose NES > 0, tend to have high positive median activity across INV-H samples and negative median activity across INV-L samples i.e. points belonging to the 4th quadrant in Fig. 3B (see also Additional file 1: Figure S4). Thus, these MRs were considered to be specific to the INV-H phenotype. Similarly, MRs whose NES < 0, generally had high positive median activity across INV-L samples and negative median activity across INV-H samples i.e. points belonging to the 2nd quadrant in Fig. 3B (see also Additional file 1: Figure S4). Thus, these MRs were considered to be specific to the INV-L phenotype.

It was noteworthy that the same MR could appear multiple times (with different colors/shapes) in Fig. 3A, B since we were showcasing the results for all ten cancers together. Additionally, we observed genes such as *SFRP2*, *ENG, BCL6B, LUM, COL1A1, and SERPINE1* were MRs for all the ten cancer subtypes (see Fig. 3C, D, Additional file 1: Table S1). Out of the 24-gene signature for invasiveness, only seven were in the list of 3,674 TRs (*SFRP4, INHBA, GREM1, FBN1, SULF1, COL1A1,* and *DCN)*. Remarkably, 2 (COL1A1 and SFRP2, orthologous to SFRP4) were MRs consistently upregulated in all the 10 INV-H cancers (see Fig. 3C). Therefore, this provides a positive validation that our approach could capture expected known genes as MRs for the INV-H phenotype.

### MR activities across prognostic cancers and enrichment analysis

**Fig. 4:**
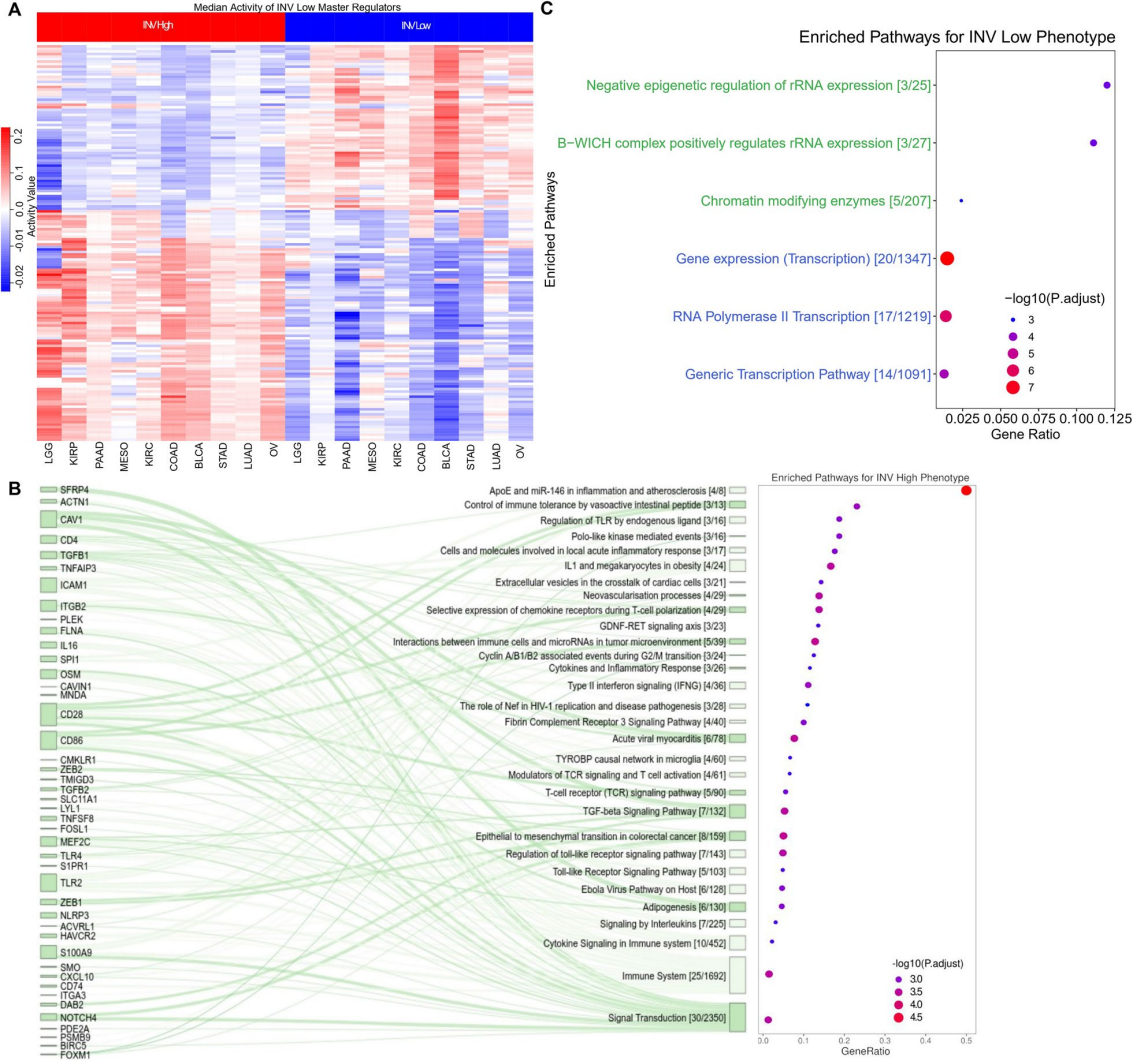
MR activities and downstream pathway enrichment analysis. **A** Top MRs specific to INV High/Low phenotype (MR in > 5 out of 10 cancers). **B** Significantly enriched pathways associated with MRs specific to INV Low. **C** Significantly enriched pathways associated with MRs specific to INV High

Once the consensus MRs were identified for each of the ten cancer types, we then estimated the MRs common across the majority of the cancer types (> 5 cancer types), resulting in a set of 156 MRs (see Additional file 1: Table S2) of which 91 MRs had median activity score significantly higher in INV-H samples when compared to INV-L samples across the ten cancer subtypes and 65 MRs had median activity score significantly higher in INV-L samples vs INV-H samples (see Additional file 1: Table S3). Therefore, these 91 and 65 MRs were considered specific to INV-H and INV-L phenotypes, respectively. The presence of shared MRs would indicate the utilization of an underlying mechanism/process by the tumor microenvironment for prognostic cancers. The median activity of these MRs across all the samples belonging to INV-H and INV-L phenotypes respectively for each of the ten prognostic cancers was depicted in Fig. 4A.

Once we had identified the MRs which were specific to INV-H (91 MRs) and INV-L (65) phenotypes respectively, across all the ten cancer types of interest, we performed downstream (enrichment) analysis using ConsensusPathDB [62]. Firstly, we considered all the 91 MRs specific to the INV-H phenotype as enriched genes and the background to be the set of all target genes (23,216 genes). We then utilized the over-expression analysis framework of ConsensusPathDB for determining gene ontology (GO) categories and enriched pathways. We identified 780 GO terms and 69 pathways that were significantly enriched (FDR-adjusted p-value ≤ 0.05) for the MRs specific to the INV-H phenotype. We demonstrated the significantly enriched GO Terms and their categories: (1) biological processes, (2) molecular functions, and (3) cellular components in Additional file 1: Fig S5A. The top biological processes included the nucleobase-containing compound biosynthetic process, regulation of the biosynthetic process, heterocycle biosynthetic process, aromatic compound biosynthetic process, organic cyclic compound biosynthetic process, cellular macromolecule biosynthetic process, cellular nitrogen compound biosynthetic process, etc.. They were primarily associated with the biosynthetic processes in the cell.

The top 30 significantly enriched pathways and associated MRs particular to the INV-H phenotype were depicted through the Sankey plot in Fig. 4B. These pathways include the Immune System (R-HSA-168256), Regulation of Toll-like Receptor signaling pathway (WP1449), Type II Interferon signaling (IFNG) (WP619), Fibrin Complement Receptor 3 signaling pathway (WP4136), Cytokine signaling in the immune system (R-HSA-1280215), Interaction between immune cells and microRNAs in the tumor microenvironment (WP4559), Epithelial to mesenchymal transition in colorectal cancer (WP4239), TGF-β signaling pathway (WP366), etc. as illustrated in Fig. 4B. Each of these pathways included at least three different MRs specific to INV-H phenotype (worse OS), thereby, suggesting higher activity of these MRs and enrichment of these pathways was detrimental to the survival of the patients categorized as INV-H across the ten cancers.

We then clustered the top 30 pathways (as well as the 69 pathways, see Additional file 1: Figure S6B) by estimating similarity in the set of enriched pathways using the extent of overlap between the MRs involved in 2 such enriched pathways. After obtaining the similarity matrix, we performed clustering of the pathways using spectral clustering [63] to differentiate the pathways into cohesive groups (5 in the case of INV-H phenotype). The pathways were color-coded by the cluster to which they belonged and the set of MRs associated with a particular pathway was depicted as an adjacency matrix (see Additional file 1: Figure S6A). Interestingly, we observed that the majority of the top significantly enriched pathways are hallmark pathways for inflammation (ApoE and miR-146 in inflammation, Cytokines, and Inflammatory Response), immune suppression (TGF-β signaling pathway, T-cell polarization), innate immune signaling (Toll-like receptor signaling, Type II Interferon signaling, Signaling by Interleukins) and precursor for metastasis (Epithelial to Mesenchymal transition), as observed in Fig. 4B and Additional file 1: Figure S6A, justifying the INV-H phenotype and its worse survival prognosis across the ten cancers of interest.

A similar analysis for the INV-H phenotype was performed for the 65 MRs specific to the INV-L phenotype. On over-expression analysis, we detected 70 GO terms and six pathways to be significantly enriched (FDR-adjusted p-value < 0.05). The significantly enriched GO terms, along with their category-level stratifications for INV-L phenotype, were showcased in Additional file 1: Figure S5b. The top GO terms included nucleic acid metabolic process, heterocycle metabolic process, nucleobase-containing compound metabolic process, cellular aromatic compound metabolic process, gene expression, etc.. They were primarily associated with the metabolic process in the cell.

The six enriched pathways particular to the INV-L phenotype include Gene Expression (Transcription) (R-HSA-74160), RNA Polymerase II Transcription (R-HSA-73857), Generic Transcription Pathway (R-HSA-212436), Negative epigenetic regulation of rRNA expression (R-HSA-5250941), B-WICH complex positively regulates rRNA expression (R-HSA-5250924) and Chromatin modifying enzymes (R-HSA-3247509). The enriched pathways were clustered into two groups as depicted in Fig. 4C, and were majorly associated with transcriptional regulation. From Fig. 4C, we observed that the maximum ratio on the x-axis reached a value of ~ 0.125, indicating that at max only one-eighth of the genes in a pathway were overexpressed. The enrichment of these pathways and the higher activities of associated MRs were beneficial for the survival of patients belonging to the INV Low group across the 10 cancers. 

Taken together, these results highlight candidate pathways such as TGF-β, Toll-like receptor signaling pathway, Epithelial to Mesenchymal transition pathway, etc., were significantly enriched in highly invasive cancers (INV-H phenotype) across multiple cancer types and can be targeted for better survival outcomes against cancer invasiveness.

### Validation of MRs for INV-N cancers & PRECOG datasets

Once we had identified the MRs which were specific to the INV-H (91 MRs) and INV-L (65 MRs) phenotype, we tried to validate these MRs in all cancers where invasiveness was not prognostic, hereby, referred as invasiveness neutral (INV-N) cancers. The goal of the validation is to showcase that MRs specific to INV-L and INV-H phenotype respectively have activity patterns in INV-N cancers similar to those in the 10 prognostically relevant cancer types. Moreover, the majority of MRs specific to INV-L and INV-H phenotypes have statisitically significant differential activity between INV-L vs INV-H samples across the invasiveness neutral cancers.

**Fig. 5:**
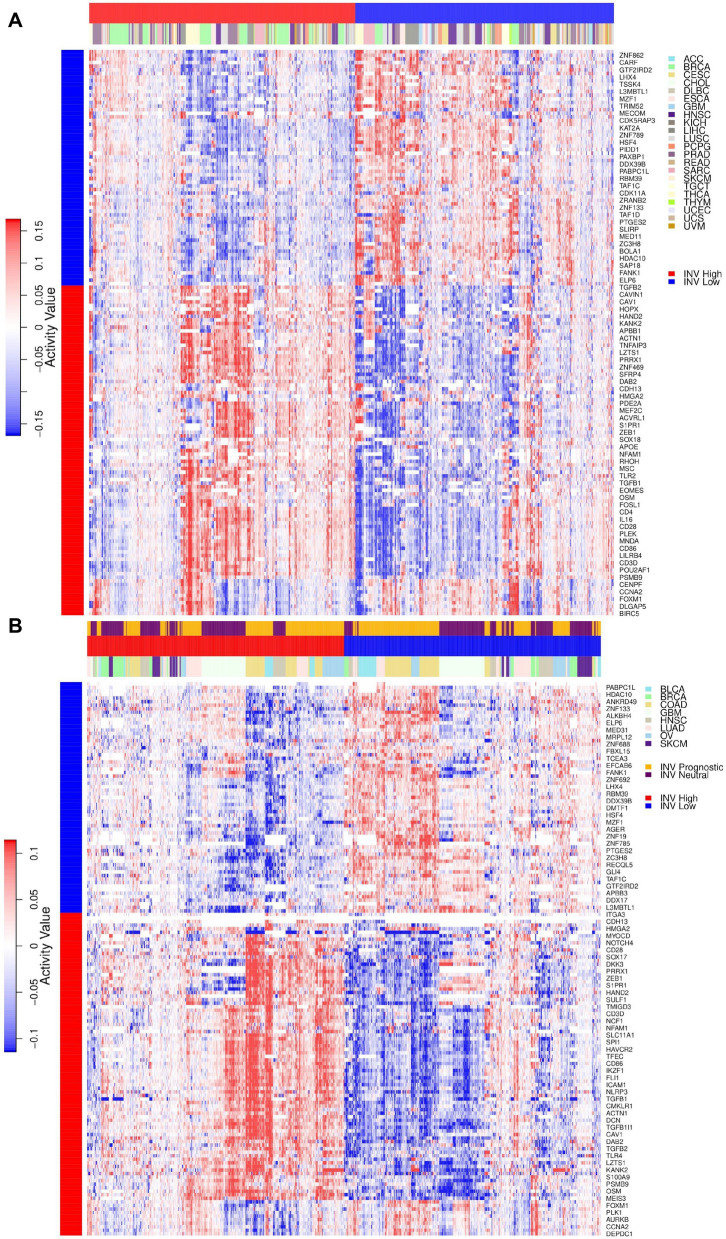
**A** Validation of INV High and INV Low specific MR activity in cancers where INV is not prognostic. **B** Independent validation of INV High and Low specific MR activity in datasets obtained from PRECOG repository. The cancer samples where invasiveness has significant association with overall survival are indicated in ‘gold’ whereas those with no significant association with overall survival are depicted in ‘purple’

To achieve this aim, we create the multi-cancer (across all INV-N cancers) MR activity matrix comprising INV-L and INV-H specific MRs as detailed in the Materials and Method section and illustrated as a heatmap in Fig. 5A. We observed that the MRs which were specific to the INV-L phenotype had predominantly high activity patterns in all INV-L samples independent of the type of cancer. In contrast, they had low activity patterns in the majority of the INV-H samples for all the 22 INV-N cancers in TCGA (see Fig. 5A and Additional file 1: Table S4 for statistical significance of differential activity comparison using Wilcoxon rank-sum test). Similarly, for the MRs associated with the INV-H phenotype, we observed that a majority of these MRs (81 out of 91) had high activities in the INV-H samples, while they had negative activities in the majority of the INV-L samples, as demonstrated in Fig. 5A (see Additional file 1: Table S4 for statistical significance of differential activity comparison using Wilcoxon rank-sum test).

We performed a similar validation on the eight datasets (BLCA, BRCA, COAD, GBM, HNSC, LUAD, OV, and SKCM cancers) obtained from the PRECOG repository. Each sample in a particular dataset was classified into INV-H or INV-L class using the 24-gene signature-derived invasiveness score. For an MR whose gene expression is not available in a particular dataset, referred as missing MR, we considered its activity value to be 0 for the INV-H and INV-L samples. We performed hierarchical clustering of the MRs specific to the INV-L phenotype based on their activity patterns in PRECOG datasets. Similar hierarchical clustering was performed for the MRs specific to the INV-H phenotype, and the two activity matrices were concatenated together along the column axis, as illustrated in Fig. 5B. We observed that the MRs which were specific to the INV-L phenotype had predominantly high activity patterns in all INV-L samples independent of the type of cancer. In contrast, they had low activity patterns in the majority of the INV-H samples in PRECOG datasets (see Fig. 5B and Additional file 1: Table S5 for statistical significance of differential activity comparison using Wilcoxon rank-sum test). Similarly, for the MRs associated with the INV-H phenotype, we observed that a majority of these MRs (80 out of 91) had high activities in the INV-H samples while they had negative activities in the majority of the INV-L samples, as demonstrated in Fig. 5B (see Additional file 1: Table S5 for statistical significance of differential activity comparison using Wilcoxon rank-sum test).

Additionally, we performed differential activity analysis between the INV-H and INV-L samples for individual cancer datasets from the PRECOG repository, to observe how many out of the 156 consensus MRs identified via TCGA dataset were differentially activated in a set of independent datasets. We identified 82, 48, 83, 78, 71, 73, 81, 45 out of the 96 INV-H specific MRs were differentially activated and 54, 20, 63, 58, 45, 56, 57, and 39 out of the 65 INV-L specific MRs were differentially activated (see Additional file 1: Fig S7 and Tables S6, S7) for BLCA, BRCA, COAD, GBM, HNSC, LUAD, OV and SKCM cancers respectively. Moreover, 13, 36, 3, 8, 16, 3, 3 and 8 MRs were missing MRs for BLCA, BRCA, COAD, GBM, HNSC, LUAD, OV and SKCM cancer respectively. Thus, we observed that more than 80% of the MRs specific to INV-H and INV-L phenotypes were significantly activated on the Wilcoxon rank-sum test) for 6 out of the 8 PRECOG validation datasets with the exception of the BRCA and SKCM cancer datasets. However, both BRCA and SKCM were cancers where the invasiveness phenotype was not prognostic (in TCGA) and in BRCA dataset 36 out of the 156 MRs were missing. This could potentially aid the observation that the majority of the consensus MRs (48 out of 96 INV-H MRs and 20 out of 65 INV-L MRs) were not differentially activated between the INV-H and INV-L samples for BRCA dataset.These two in-silico validations confirm that the MRs which we identified were specific to the INV-H and INV-L phenotypes, respectively and the MRs specific to the INV-H phenotype (worse OS) would likely be involved in inflammatory and immune exclusion functions. Thus, the enriched pathways associated with these MRs could potentially represent molecular mechanisms driving cancer invasiveness and could be targeted to design better therapeutic strategies to tackle cancer invasiveness.

## Discussion

The estimation of TR activities from RNA-Seq data was a recent phenomenon and has attracted attention in cancer research [16, 58, 64]. While multiple techniques [16, 24] have been used to estimate TR activity profiles based on varying notions of TR regulons, the common consensus was that mRNA levels of target genes of a TR could be used to identify its activity profile. Moreover, TRs that were differentially activated w.r.t a phenotype of interest, i.e., MRs could be considered prognostic markers while revealing novel mechanisms associated with the tumor microenvironment. However, the exploration of MRs as therapeutic targets, alone or in combination with other biomarkers was a recent occurrence [16, 24, 58].

Here, we designed and applied 4 different MRA pipelines using the TCGA RNA-Seq data to discover differentially activated TRs (MRs) w.r.t the invasiveness phenotype (INV-H vs INV-L). We took a consensus of the MRs identified by these varied MRA pipelines for our goal of identifying key driver MRs for the INV-H phenotype associated with worse survival outcomes. Our network-based framework led to the discovery of 91 MRs specific to the INV-H phenotype and 65 MRs specific to the INV-L phenotype. Downstream analysis of the MRs specific to INV-H using ConsensusPathDB showed significant enrichment of pathways that were the hallmark of an inflammatory immune response.

Since, the primary goal of our work was to identify key driver genes and their associated mechanisms for higher cancer invasiveness (INV-H) leading to worse survival, downstream analysis of MRs specific to INV-H phenotype using ConsensusPathDB resulted in the enrichment of pathways such as local acute inflammatory response which is known to play a decisive role at different stages of tumor development including initiation, promotion, invasion, and metastasis [65]. MRs mediate pathways such as toll-like receptor signaling. *TLR2*, *TLR4,* and inflammasome inducing MR, *NLRP3* [66] can lead to tumor progression via the production of inflammatory cytokines (*IL6*, *IL16*), increased cell proliferation, and resistance to apoptosis (*TNFAIP3*) [66, 67]. Moreover, enrichment of pathways such as epithelial to mesenchymal transition mediated by INV-H specific MRs: *NOTCH3*, *NOTCH4*, *ZEB1*, *ZEB2, TGFB1, TGFB2,* and extracellular matrix organization, ECM proteoglycans through activation of MRs: *DCN*, *TGFB1*, *TGFB2*, *ITGB2*, *ITGA3*, *ACTN1,* and *ICAM1* are hallmarks of cancer metastasis [68] and stemness [69] respectively.

Similarly, TGF-β (TGFB1 and TGFB2) is a known immune suppressor [70]. Its high activation in INV-H samples of the ten prognostic cancers, along with enrichment of T-cell receptor (TCR) signaling and selective expression of chemokine receptors during T-cell polarization (involving MRs: *CD4*, *CD28*, *TGFB1*, and *TGFB2*) suggests the occurrence of the phenomenon, such as immune exhaustion, leading to poor survival rates in these INV-H tumor samples. This observation agrees with very recent data in mice demonstrating that blocking TGFB1 overcomes resistance to immune checkpoint inhibition [71]. The list of MRs generated by our analysis might be exploited for future targeted therapy combinations aimed at overcoming immune exhaustion or tumorigenesis, therefore, potentially extending the benefit of immunotherapy.

Our results demonstrate that TR activity profiles inferred from RNA-Seq data using RGBM + FGSEA, RGBM + GSVA, RGBM + Viper, and ARACNE + Viper MRA pipelines can be used to discover key MRs associated with the cancer invasiveness phenotype. In-silico validation of this consensus MRs was performed in INV-N cancers and a set of 8 different datasets was collected from the PRECOG repository, suggesting that these MRs can be used as promising therapeutic markers.

### Supplementary Information

**Additional file 1: Figure S1: **Kaplan-Meier plot highlights the difference in survival between the INV High vs INV Low groups for the 10 cancers of interest. Here OV is Ovarian Cancer, LUAD is Lung Adenocarcinoma, STAD is Stomach Adenocarcinoma, BLCA is Bladder Urothelial Carcinoma, COAD is Colon Adenocarcinoma, KIRC is Kidney Renal Cell Carcinoma, MESO is Mesothelioma, PAAD is Pancreatic Adenocarcinoma, KIRP is Kidney Renal Papillary Cell Carcinoma and LGG is Low Grade Gliomas. **Figure S2: **Quantile-Normalized and log2 transformed gene expression profiles for BLCA tumor samples. **Figure S3: **Activity profiles of transcriptional regulators follow a normal distribution for a particular cancer. **Figure S4: **Here we highlight that when the normalized enrichment scores (NES) for TRs are positive then, these TRs have high positive activity in INV-H samples and high negative activity in INV-L samples. Thus, TRs with positive NES scores are more specific to the INV-H phenotype. Similarly, when the NES are negative for TRs then, these TRs have high positive activity in INV-L samples and high negative activity in INV-H samples. Thus, TRs with negative NES are more specific to the INV-L phenotype (p-value < 1e-5). **Figure S5: **GO Terms including Biological Processes (b), Cellular Components (c ), Molecular Functions (m) which are significantly enriched when performing over-expression analysis of common MRs for INV-H and INV-L phenotype respectively. **Figure S6**: The top enriched pathways obtained by over-expression analysis for the top MRs peculiar to INV High phenotype are highlighted here. The pathways are clustered and color-coded according to their respective clusters. **Figure S7**: Heatmaps depicting the MR activity of the MRs specific to INV-H and INV-L phenotypes (based on the 10 prognostic cancers) and present in each of the 8 PRECOG validation datasets. **Table S1**: MRs common across all the 10 cancer types of interest and specific to INV-H phenotype (Mean FC > 0). **Table S2**: List of master regulators (differentially activated TRs) common across 6 or more prognostic cancer subtypes. We showcase the cancers for which these MRs are differentially active. **Table S3**: List of 156 significant MRs common across the majority of the prognostic cancers and are ranked based on fold change (FC) between activities in INV-H vs activities in INV-L samples across all the 10 cancers using Wilcoxon rank-sum test. **Table S4**: List of 156 MRs specific to INV-L and INV-H phenotype and their activity profile for the set of 22 INV Neutral cancers. The significance (155 out of 156) of difference in activities in INV-H vs INV-L cancer samples is highlighted using Wilcoxon rank-sum test. **Table S5**: List of 156 MRs specific to INV-L and INV-H phenotype and their activity profile for the set of 8 PRECOG datasets. The significance (153 out of 156) of the difference in activities in INV-H vs INV-L cancer samples is highlighted using the Wilcoxon rank-sum test. **Table S6**: Differentially activated MRs (out of 156 MRs) in the PRECOG cancer datasets for BLCA, BRCA, COAD and GBM cancer types. **Table S7**: Differentially activated MRs (out of 156 MRs) in the PRECOG cancer datasets for HNSC, LUAD, OV and SKCM cancer types.

## Data Availability

All the data and code are available at https://github.com/raghvendra5688/INV_Analysis
