# Supplementary material for: Pancancer network analysis reveals key master regulators for cancer invasiveness
Source: J Transl Med. 2023 Aug 20;21:558. doi: 10.1186/s12967-023-04435-6 (PMC10440887; doi:10.1186/s12967-023-04435-6)
Supplement: Supplementary file 1 — Additional file 1: Figure S1: Kaplan-Meier plot highlights the difference in survival between the INV High vs INV Low groups for the 10 cancers of interest. Here OV is Ovarian Cancer, LUAD is Lung Adenocarcinoma, STAD is Stomach Adenocarcinoma, BLCA is Bladder Urothelial Carcinoma, COAD is Colon Adenocarcinoma, KIRC is Kidney Renal Cell Carcinoma, MESO is Mesothelioma, PAAD is Pancreatic Adenocarcinoma, KIRP is Kidney Renal Papillary Cell Carcinoma and LGG is Low Grade Gliomas. Figure S2: Quantile-Normalized and log2 transformed gene expression profiles for BLCA tumor samples. Figure S3: Activity profiles of transcriptional regulators follow a normal distribution for a particular cancer. Figure S4: Here we highlight that when the normalized enrichment scores (NES) for TRs are positive then, these TRs have high positive activity in INV-H samples and high negative activity in INV-L samples. Thus, TRs with positive NES scores are more specific to the INV-H phenotype. Similarly, when the NES are negative for TRs then, these TRs have high positive activity in INV-L samples and high negative activity in INV-H samples. Thus, TRs with negative NES are more specific to the INV-L phenotype (p-value < 1e-5). Figure S5: GO Terms including Biological Processes (b), Cellular Components (c ), Molecular Functions (m) which are significantly enriched when performing over-expression analysis of common MRs for INV-H and INV-L phenotype respectively. Figure S6: The top enriched pathways obtained by over-expression analysis for the top MRs peculiar to INV High phenotype are highlighted here. The pathways are clustered and color-coded according to their respective clusters. Figure S7: Heatmaps depicting the MR activity of the MRs specific to INV-H and INV-L phenotypes (based on the 10 prognostic cancers) and present in each of the 8 PRECOG validation datasets. Table S1: MRs common across all the 10 cancer types of interest and specific to INV-H phenotype (Mean FC > 0). Table S2: List of [file 12967_2023_4435_MOESM1_ESM.pdf]

Additional file

Pancancer Network Analysis reveals key Master Regulators for Cancer Invasiveness

Mall et al

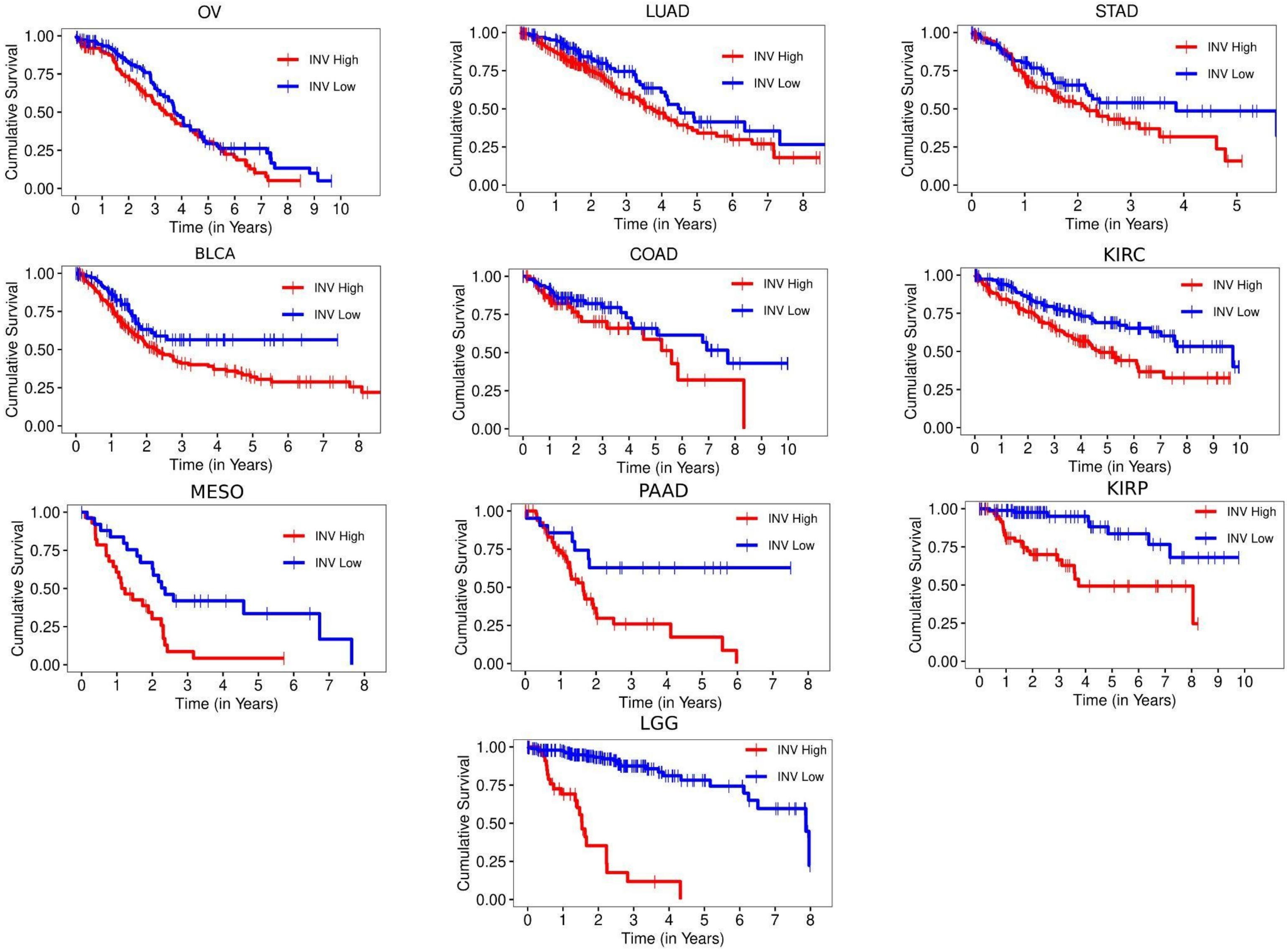

Figure S1: Kaplan-Meier plot highlights the difference in survival between the INV High vs INV Low groups for the 10 cancers of interest. Here OV is Ovarian Cancer, LUAD is Lung Adenocarcinoma, STAD is Stomach Adenocarcinoma, BLCA is Bladder Urothelial Carcinoma, COAD is Colon Adenocarcinoma, KIRC is Kidney Renal Cell Carcinoma, MESO is Mesothelioma, PAAD is Pancreatic Adenocarcinoma, KIRP is Kidney Renal Papillary Cell Carcinoma and LGG is Low Grade Gliomas.

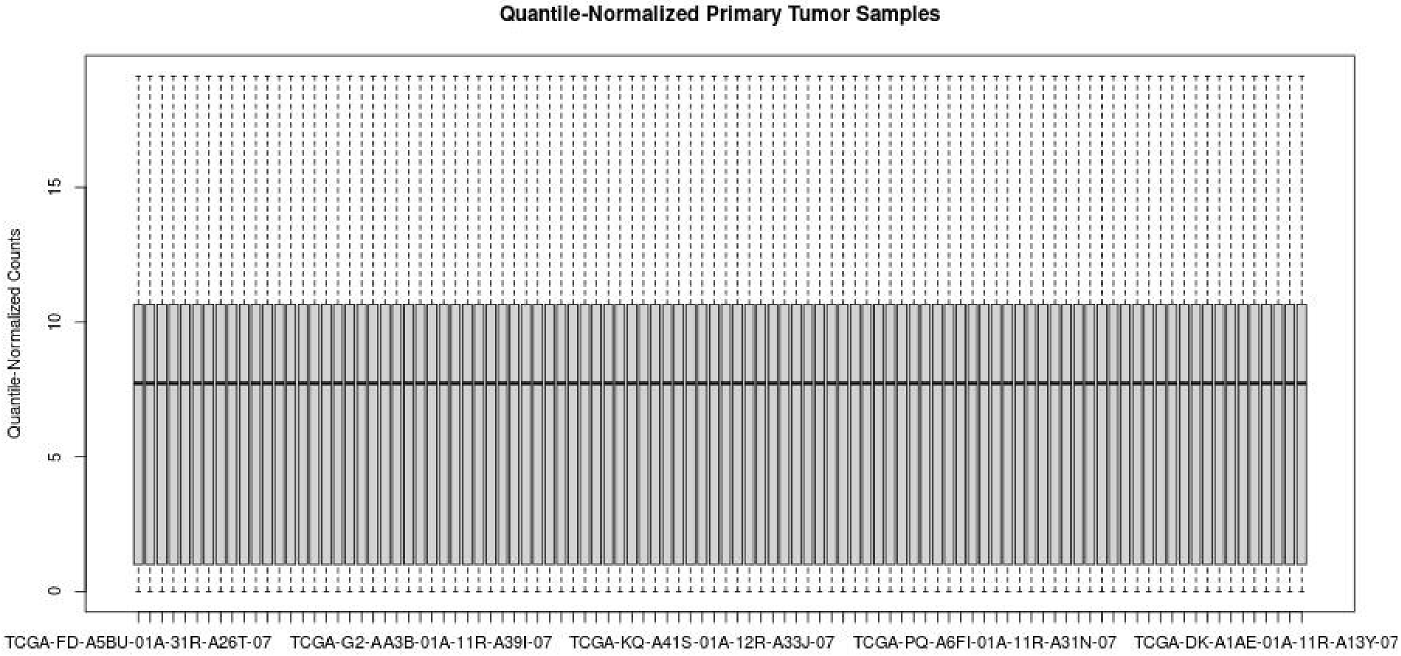

Figure S2: Quantile-Normalized and log2 transformed gene expression profiles for BLCA tumor samples.

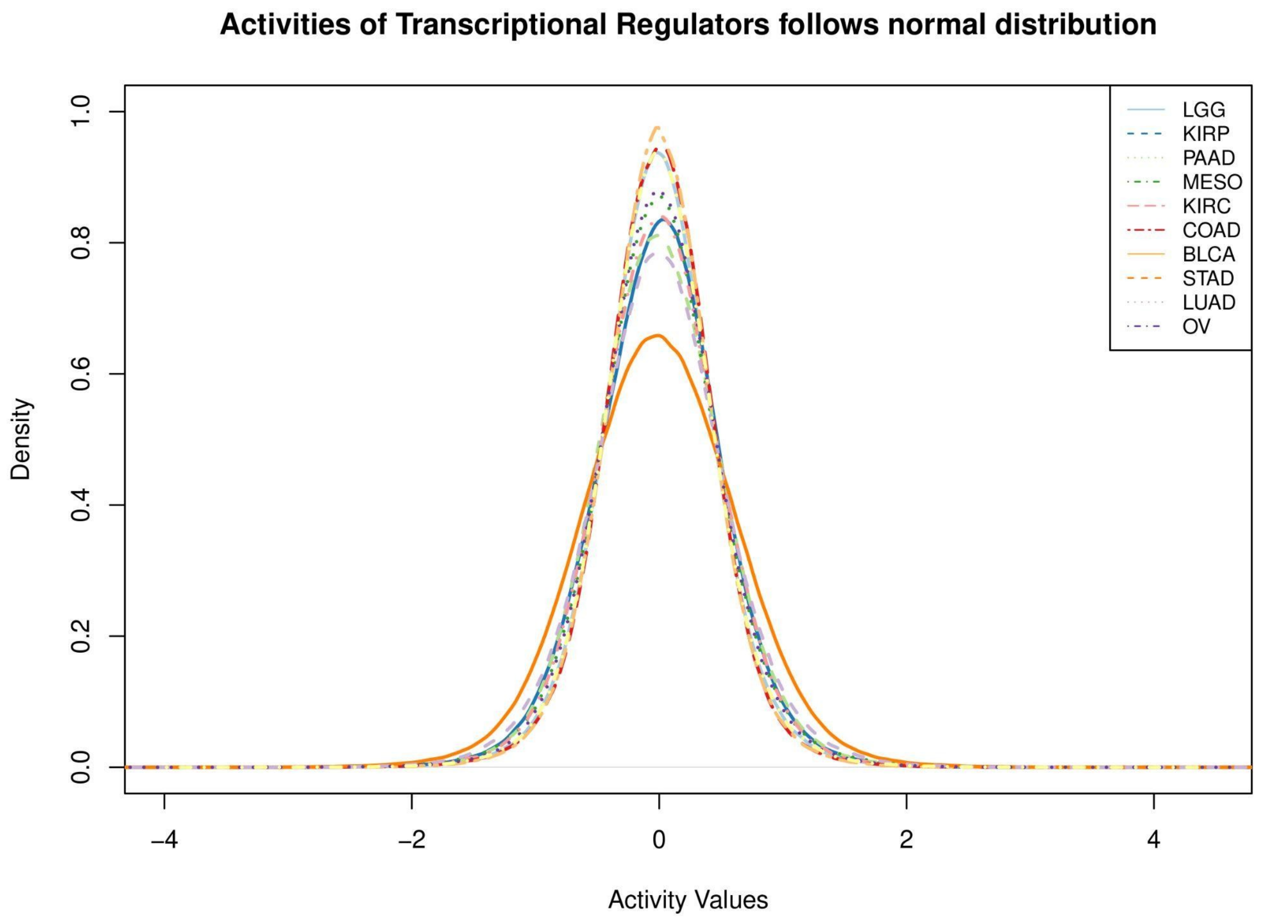

Figure S3: Activity profiles of transcriptional regulators follow a normal distribution for a particular cancer.

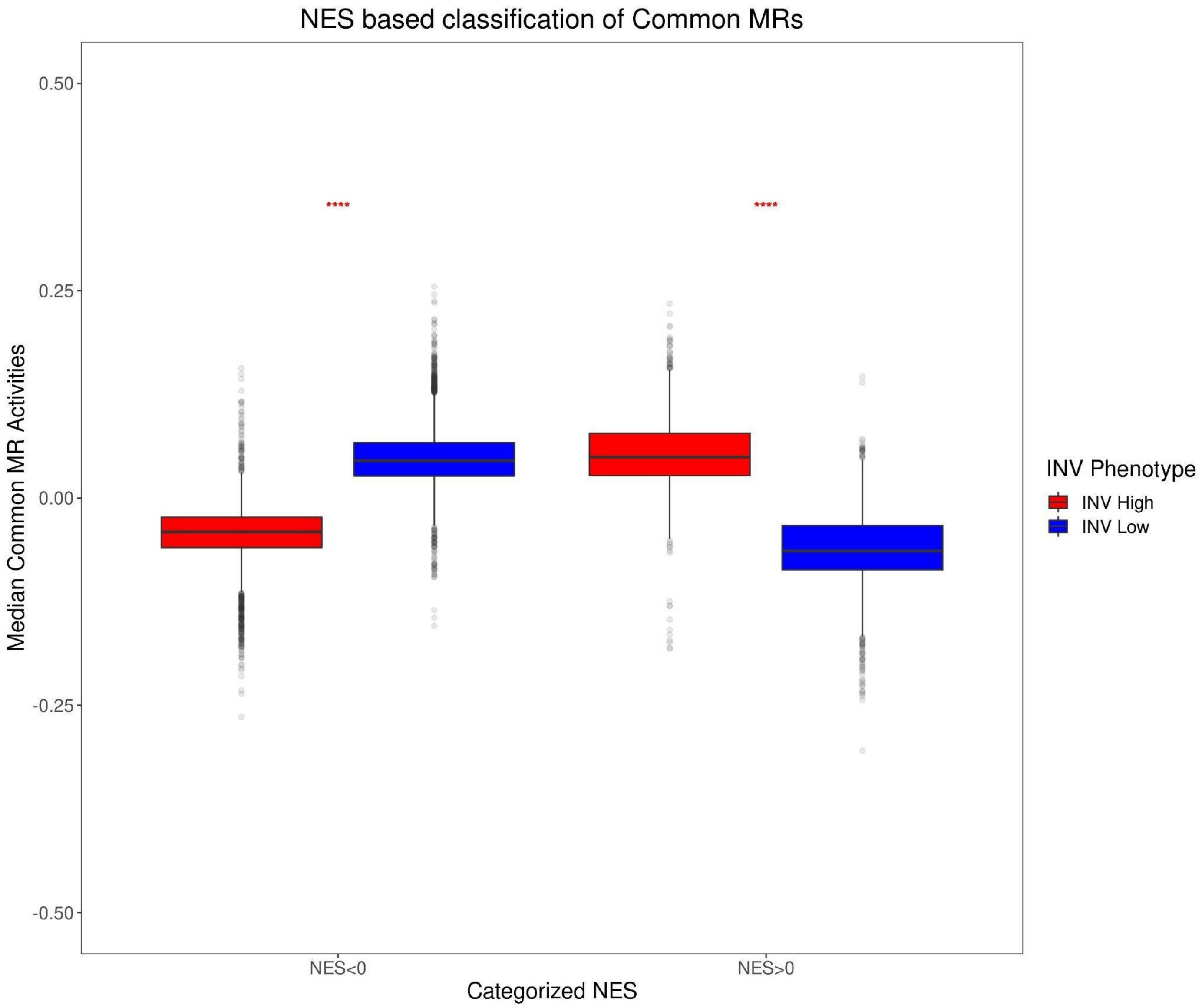

Figure S4: Here we highlight that when the normalized enrichment scores (NES) for TRs are positive then, these TRs have high positive activity in INV-H samples and high negative activity in INV-L samples. Thus, TRs with positive NES scores are more specific to the INV-H phenotype. Similarly, when the NES are negative for TRs then, these TRs have high positive activity in INV-L samples and high negative activity in INV-H samples. Thus, TRs with negative NES are more specific to the INV-L phenotype (p-value < 1e-5).

**MR**	**Statistic**	**P-value**	**Mean INV-H**	**Mean INV-L**	**Mean FC**	**P-adjust**
COL1A1	100	0.00018	0.069	-0.081	0.15	0.00037
SERPINE1	100	0.00018	0.066	-0.079	0.144	0.00037
LUM	100	0.00018	0.068	-0.069	0.136	0.00037
SFRP2	92	0.00171	0.058	-0.074	0.131	0.00171
ENG	96	0.00058	0.056	-0.052	0.108	0.0007

BCL6B	99	0.00025	0.051	-0.048	0.1	0.00037

Table S1: MRs common across all the 10 cancer types of interest and specific to INV-H phenotype (Mean FC > 0).

**MR**	**Prognostic Cancer Subtypes**	**Total Cancer Subtypes**
SPI1	LGG KIRP PAAD KIRC COAD BLCA STAD LUAD OV	9
ITGB2	LGG KIRP PAAD KIRC COAD BLCA STAD LUAD OV	9
DCN	LGG KIRP PAAD KIRC COAD BLCA STAD LUAD OV	9
TFEC	LGG KIRP PAAD KIRC COAD BLCA STAD LUAD OV	9
CD4	LGG KIRP PAAD KIRC COAD BLCA STAD LUAD OV	9
TNFSF8	LGG KIRP PAAD KIRC COAD BLCA STAD LUAD OV	9
CD86	LGG KIRP PAAD KIRC COAD BLCA STAD LUAD OV	9
HCK	LGG KIRP PAAD KIRC COAD BLCA STAD LUAD OV	9
PLEK	LGG KIRP PAAD KIRC COAD BLCA STAD LUAD OV	9
NFAM1	LGG KIRP PAAD KIRC COAD BLCA STAD LUAD OV	9
SFRP4	LGG KIRP PAAD MESO COAD BLCA STAD LUAD OV	9
IL6	LGG KIRP PAAD KIRC COAD BLCA STAD LUAD OV	9
HSF4	LGG KIRP PAAD MESO KIRC COAD BLCA STAD LUAD	9
TRIM52	LGG KIRP PAAD MESO KIRC COAD BLCA STAD LUAD	9
CDH13	LGG KIRP PAAD MESO KIRC COAD STAD LUAD OV	9
TGFB1	LGG KIRP PAAD MESO KIRC COAD STAD LUAD OV	9
PRRX1	KIRP PAAD MESO KIRC COAD BLCA STAD LUAD OV	9
GREM1	KIRP PAAD MESO KIRC COAD BLCA STAD LUAD OV	9
TGFB1I1	KIRP PAAD MESO KIRC COAD BLCA STAD LUAD OV	9
MEIS3	KIRP PAAD MESO KIRC COAD BLCA STAD LUAD OV	9
CAVIN1	LGG PAAD KIRC COAD BLCA STAD LUAD OV	8
LILRB4	LGG PAAD KIRC COAD BLCA STAD LUAD OV	8
MECOM	LGG KIRP MESO KIRC COAD BLCA STAD LUAD	8

NLRP3	LGG PAAD KIRC COAD BLCA STAD LUAD OV	8
TMIGD3	LGG PAAD KIRC COAD BLCA STAD LUAD OV	8
CXCL10	LGG KIRP PAAD COAD BLCA STAD LUAD OV	8
FLI1	LGG KIRP PAAD KIRC COAD BLCA STAD OV	8
DAB2	LGG PAAD KIRC COAD BLCA STAD LUAD OV	8
RHOH	LGG KIRP PAAD KIRC COAD BLCA LUAD OV	8
VGLL3	LGG KIRP PAAD KIRC COAD STAD LUAD OV	8
ZNF785	LGG KIRP PAAD MESO KIRC BLCA STAD LUAD	8
BOLA1	LGG KIRP PAAD MESO KIRC COAD BLCA STAD	8
ZNF692	LGG KIRP KIRC COAD BLCA STAD LUAD OV	8
CARF	LGG KIRP KIRC COAD BLCA STAD LUAD OV	8
MED11	LGG KIRP PAAD MESO KIRC COAD BLCA LUAD	8
SMO	LGG KIRP PAAD COAD BLCA STAD LUAD OV	8
SARNP	LGG KIRP PAAD KIRC COAD BLCA STAD LUAD	8
MZF1	LGG KIRP PAAD KIRC COAD BLCA STAD LUAD	8
HOPX	LGG KIRP PAAD KIRC COAD STAD LUAD OV	8
ERG	KIRP MESO KIRC COAD BLCA STAD LUAD OV	8
ZNF789	KIRP PAAD KIRC COAD BLCA STAD LUAD OV	8
SULF1	KIRP PAAD KIRC COAD BLCA STAD LUAD OV	8
GTF2IRD2	KIRP PAAD KIRC COAD BLCA STAD LUAD OV	8
ZNF862	KIRP PAAD KIRC COAD BLCA STAD LUAD OV	8
CYR61	KIRP PAAD MESO COAD BLCA STAD LUAD OV	8
GTF2IRD2B	KIRP PAAD KIRC COAD BLCA STAD LUAD OV	8
ZNF469	PAAD MESO KIRC COAD BLCA STAD LUAD OV	8
LZTS1	PAAD MESO KIRC COAD BLCA STAD LUAD OV	8
ACTN1	LGG PAAD MESO KIRC BLCA STAD LUAD	7
TGFB2	LGG KIRP PAAD MESO KIRC BLCA STAD	7
CAV1	LGG KIRP PAAD COAD BLCA STAD OV	7

FANK1	LGG KIRP MESO KIRC COAD LUAD OV	7
HAVCR2	LGG PAAD COAD BLCA STAD LUAD OV	7
ACVRL1	LGG PAAD MESO KIRC BLCA STAD OV	7
IKZF1	LGG KIRP PAAD KIRC COAD BLCA OV	7
DEPDC1	LGG KIRP MESO KIRC COAD STAD LUAD	7
SLC11A1	LGG PAAD COAD BLCA STAD LUAD OV	7
CENPF	LGG KIRP MESO KIRC COAD STAD LUAD	7
FOXM1	LGG KIRP MESO KIRC COAD STAD LUAD	7
BIRC5	LGG KIRP MESO KIRC COAD STAD LUAD	7
TOP2A	LGG KIRP MESO KIRC COAD STAD LUAD	7
CD3D	LGG KIRP PAAD KIRC COAD BLCA OV	7
IL16	LGG KIRP PAAD KIRC COAD BLCA OV	7
FOSL1	LGG KIRP PAAD KIRC BLCA LUAD OV	7
ICAM1	LGG PAAD KIRC COAD BLCA STAD OV	7
MNDA	LGG KIRP PAAD COAD BLCA STAD OV	7
TSSK4	LGG KIRP KIRC COAD BLCA STAD LUAD	7
APBB3	LGG KIRP KIRC COAD BLCA STAD LUAD	7
ZEB2	LGG PAAD COAD BLCA STAD LUAD OV	7
ZNF337	LGG KIRP KIRC COAD BLCA STAD LUAD	7
HDAC10	LGG PAAD KIRC COAD BLCA STAD LUAD	7
ZNF23	LGG KIRP KIRC COAD BLCA STAD LUAD	7
S1PR1	LGG KIRP MESO COAD BLCA STAD OV	7
ZNF276	LGG KIRP KIRC COAD BLCA STAD LUAD	7
ZC3H8	LGG KIRP PAAD KIRC COAD STAD LUAD	7
STK36	LGG KIRP KIRC COAD BLCA LUAD OV	7
GLI4	LGG KIRP MESO KIRC COAD BLCA LUAD	7
MYOCD	LGG KIRP KIRC COAD BLCA STAD OV	7
ZNF169	LGG KIRP KIRC COAD BLCA STAD LUAD	7

ZNF354B	LGG KIRP KIRC COAD BLCA STAD LUAD	7
ZNF688	LGG KIRP PAAD MESO KIRC COAD BLCA	7
ZRANB2	LGG KIRP COAD BLCA STAD LUAD OV	7
LHX4	LGG KIRP KIRC COAD BLCA STAD LUAD	7
NOTCH3	KIRP PAAD MESO KIRC COAD STAD LUAD	7
AGER	KIRP KIRC COAD BLCA STAD LUAD OV	7
RBM39	KIRP KIRC COAD BLCA STAD LUAD OV	7
TAF1C	KIRP KIRC COAD BLCA STAD LUAD OV	7
LPIN3	KIRP MESO KIRC COAD BLCA STAD LUAD	7
SOX18	KIRP MESO KIRC COAD BLCA STAD LUAD	7
OSM	KIRP PAAD KIRC COAD BLCA STAD OV	7
ALKBH4	KIRP PAAD KIRC COAD BLCA STAD OV	7
ZNF19	KIRP PAAD KIRC COAD BLCA LUAD OV	7
MRPL12	KIRP PAAD MESO KIRC COAD BLCA STAD	7
KANK2	PAAD MESO KIRC COAD BLCA STAD LUAD	7
MEF2C	PAAD MESO KIRC COAD BLCA STAD OV	7
ZEB1	PAAD KIRC COAD BLCA STAD LUAD OV	7
DKK3	PAAD MESO KIRC COAD BLCA STAD LUAD	7
MSC	PAAD KIRC COAD BLCA STAD LUAD OV	7
FLNA	LGG PAAD KIRC COAD BLCA STAD	6
CCNL2	LGG KIRP COAD BLCA STAD LUAD	6
TCEA3	LGG KIRP PAAD KIRC COAD LUAD	6
ITGA3	LGG KIRP PAAD KIRC LUAD OV	6
NCF1	LGG PAAD COAD BLCA LUAD OV	6
CD74	LGG KIRP PAAD COAD BLCA OV	6
LYL1	LGG KIRP KIRC BLCA STAD OV	6
CDC45	LGG MESO KIRC COAD STAD LUAD	6
CCNA2	LGG MESO KIRC COAD STAD LUAD	6

DLGAP5	LGG MESO KIRC COAD STAD LUAD	6
CD28	LGG PAAD KIRC COAD BLCA OV	6
AURKB	LGG MESO KIRC COAD STAD LUAD	6
TLR2	LGG PAAD KIRC COAD STAD OV	6
PYHIN1	LGG KIRP PAAD COAD BLCA OV	6
PSMB9	LGG PAAD COAD BLCA LUAD OV	6
EOMES	LGG KIRP PAAD COAD BLCA OV	6
S100A9	LGG KIRP PAAD COAD BLCA LUAD	6
PLK1	LGG MESO KIRC COAD STAD LUAD	6
ATP8B1	LGG MESO KIRC BLCA STAD LUAD	6
L3MBTL1	LGG KIRP KIRC BLCA LUAD OV	6
DDX17	LGG KIRP KIRC BLCA STAD LUAD	6
CENPT	LGG KIRP KIRC COAD BLCA LUAD	6
APBB1	LGG KIRP KIRC COAD BLCA STAD	6
KAT2A	LGG KIRP KIRC COAD BLCA LUAD	6
ZNF133	LGG KIRP PAAD BLCA STAD LUAD	6
PDE2A	LGG KIRP COAD BLCA STAD LUAD	6
NOTCH4	LGG KIRP MESO COAD STAD OV	6
FOXC2	LGG KIRP MESO KIRC BLCA OV	6
GATAD1	LGG KIRP MESO KIRC COAD LUAD	6
FBXL15	LGG KIRP PAAD KIRC COAD LUAD	6
MXD3	LGG KIRP COAD BLCA STAD LUAD	6
TNFAIP3	LGG KIRP PAAD BLCA LUAD OV	6
CDK11A	LGG KIRP COAD BLCA STAD LUAD	6
HMGA2	LGG PAAD KIRC BLCA LUAD OV	6
MSRB2	LGG KIRP MESO KIRC COAD LUAD	6
APOE	LGG PAAD KIRC COAD BLCA STAD	6
TAF1D	LGG COAD BLCA STAD LUAD OV	6

SAP18	LGG KIRP PAAD KIRC COAD LUAD	6
PTGES2	LGG KIRP PAAD KIRC COAD STAD	6
DMTF1	KIRP KIRC COAD BLCA STAD LUAD	6
DDX39B	KIRP KIRC COAD BLCA STAD LUAD	6
PABPC1L	KIRP COAD BLCA STAD LUAD OV	6
CDK5RAP3	KIRP KIRC COAD BLCA STAD LUAD	6
CMKLR1	KIRP KIRC COAD BLCA STAD OV	6
PNN	KIRP COAD BLCA STAD LUAD OV	6
POU2AF1	KIRP PAAD KIRC COAD BLCA LUAD	6
ANKRD49	KIRP PAAD KIRC COAD BLCA STAD	6
SOX17	KIRP MESO KIRC COAD BLCA STAD	6
PAXBP1	KIRP KIRC COAD BLCA STAD LUAD	6
EFCAB6	KIRP PAAD MESO KIRC LUAD OV	6
RECQL5	KIRP KIRC COAD BLCA STAD LUAD	6
ELP6	KIRP PAAD COAD BLCA STAD LUAD	6
MED31	KIRP PAAD MESO COAD BLCA LUAD	6
TLR4	KIRP PAAD KIRC STAD LUAD OV	6
SLIRP	KIRP PAAD KIRC COAD BLCA STAD	6
BNC2	PAAD COAD BLCA STAD LUAD OV	6
HAND2	PAAD KIRC COAD BLCA STAD OV	6
ZNF423	PAAD KIRC COAD STAD LUAD OV	6
PIDD1	PAAD KIRC COAD STAD LUAD OV	6

Table S2: List of master regulators (differentially activated TRs) common across 6 or more prognostic cancer subtypes. We showcase the cancers for which these MRs are differentially active.

**MR**	**Median FC**	**FDR-adjusted P-value**
PRRX1	0.14	3.59088E-199

SFRP4	0.13	2.11824E-164
IL6	0.129	1.2632E-187
SULF1	0.126	1.89773E-186
GREM1	0.124	1.92755E-175
ACTN1	0.119	2.27279E-178
HAND2	0.117	5.80894E-94
CYR61	0.116	1.56904E-114
CAV1	0.114	2.78075E-144
CD4	0.113	1.99725E-146
DCN	0.113	5.9343E-140
TGFB1	0.113	3.09548E-151
TGFB1I1	0.112	3.61244E-173
TNFAIP3	0.111	2.54043E-129
ICAM1	0.108	3.30611E-143
ITGB2	0.108	3.2079E-136
IKZF1	0.106	2.04186E-130
PLEK	0.106	2.94464E-134
FLI1	0.105	3.92372E-133
FLNA	0.105	2.7219E-155
ZNF469	0.103	8.61599E-140
RHOH	0.102	1.19118E-131
IL16	0.1	1.90517E-115
NFAM1	0.1	6.27254E-110
VGLL3	0.1	3.19178E-123
SPI1	0.099	1.27432E-116
HCK	0.098	2.04889E-125
OSM	0.098	8.30795E-137

CAVIN1	0.096	1.90517E-115
LZTS1	0.096	8.24837E-119
LILRB4	0.095	6.38461E-129
MNDA	0.094	8.94768E-118
CD28	0.093	1.65997E-89
KANK2	0.093	5.6825E-66
CD86	0.092	6.51811E-111
APOE	0.09	1.66803E-111
CMKLR1	0.089	2.22728E-90
HOPX	0.089	3.43635E-77
MEIS3	0.088	2.10216E-121
ZEB2	0.088	9.04445E-105
PYHIN1	0.087	1.99051E-92
TMIGD3	0.087	3.16117E-97
MSC	0.086	5.71549E-107
TGFB2	0.086	1.10253E-83
CD3D	0.085	2.05457E-96
SLC11A1	0.084	1.85649E-114
LYL1	0.083	6.10771E-74
NCF1	0.083	1.11961E-101
BNC2	0.082	2.37692E-104
TNFSF8	0.082	3.26604E-93
SOX18	0.081	1.07978E-80
FOSL1	0.079	2.31673E-109
MEF2C	0.079	2.4913E-70
TLR4	0.079	5.35989E-96
S1PR1	0.078	1.70724E-77
CDH13	0.076	1.93291E-76

FOXC2	0.076	7.06834E-60
NOTCH3	0.075	1.33169E-64
EOMES	0.074	1.05653E-83
TLR2	0.073	2.59306E-79
ZEB1	0.072	5.2941E-60
DKK3	0.071	1.26873E-57
NLRP3	0.071	9.61725E-98
ACVRL1	0.069	3.05705E-73
HAVCR2	0.069	1.59516E-69
ERG	0.068	1.32955E-61
S100A9	0.067	3.68347E-87
TFEC	0.066	2.67477E-55
POU2AF1	0.064	1.85099E-66
SMO	0.06	1.81987E-52
CXCL10	0.056	4.04738E-59
HMGA2	0.055	1.61796E-35
CD74	0.053	1.59335E-38
ITGA3	0.052	9.7305E-62
SOX17	0.05	6.52188E-31
MYOCD	0.048	5.55969E-24
DAB2	0.046	8.55049E-34
NOTCH4	0.046	1.77407E-39
PDE2A	0.045	5.84218E-24
PSMB9	0.043	1.35109E-33
CCNA2	0.041	1.66133E-22
DLGAP5	0.04	3.21625E-20
DEPDC1	0.034	3.70209E-15
CENPF	0.033	3.7646E-18

PLK1	0.033	2.832E-17
BIRC5	0.026	1.05808E-12
FOXM1	0.026	3.47683E-13
AURKB	0.025	5.02519E-12
CDC45	0.017	7.26553E-05
APBB1	0.011	0.00107077
TOP2A	0.011	0.0427786
ATP8B1	-0.012	7.65375E-05
MECOM	-0.028	3.49912E-20
SLIRP	-0.029	5.2527E-20
MRPL12	-0.03	1.9839E-14
SAP18	-0.031	1.08252E-17
ZNF862	-0.031	7.7636E-15
MSRB2	-0.035	2.14344E-11
DMTF1	-0.041	6.47611E-39
MXD3	-0.044	3.12443E-21
MED31	-0.047	5.68006E-29
CCNL2	-0.05	2.79861E-37
ZNF688	-0.05	1.54615E-43
ALKBH4	-0.051	1.16661E-39
PTGES2	-0.051	1.20333E-34
GATAD1	-0.052	2.93727E-39
ZRANB2	-0.054	3.79038E-49
CENPT	-0.056	6.76405E-54
DDX17	-0.056	1.88148E-55
ELP6	-0.057	4.09106E-53
TAF1D	-0.058	4.14829E-56
ZNF789	-0.058	8.23431E-54

RBM39	-0.059	5.27432E-51
ZNF354B	-0.059	3.37682E-55
LPIN3	-0.06	9.20356E-47
PABPC1L	-0.06	3.0138E-57
TCEA3	-0.06	1.69945E-44
RECQL5	-0.061	6.17828E-61
STK36	-0.063	2.19279E-65
ZNF337	-0.063	5.49267E-75
TSSK4	-0.064	2.05381E-88
DDX39B	-0.065	1.16542E-74
MED11	-0.065	1.04371E-89
BOLA1	-0.066	2.41146E-58
PNN	-0.066	2.91248E-72
HDAC10	-0.067	3.68689E-73
PAXBP1	-0.067	2.09283E-68
FBXL15	-0.068	1.49787E-91
PIDD1	-0.068	6.25217E-52
GLI4	-0.071	3.03959E-67
ZNF276	-0.071	7.98378E-85
MZF1	-0.072	2.88872E-80
CDK11A	-0.073	2.67798E-52
CDK5RAP3	-0.073	2.31819E-90
ANKRD49	-0.074	9.32838E-67
FANK1	-0.074	2.01155E-67
SARNP	-0.074	2.83815E-71
TAF1C	-0.074	3.05395E-87
ZNF133	-0.075	7.66943E-69
ZNF785	-0.075	3.95633E-88

APBB3	-0.076	2.29993E-113
ZNF23	-0.076	3.35178E-83
ZC3H8	-0.078	3.00048E-87
CARF	-0.079	1.06663E-91
HSF4	-0.079	1.74883E-102
KAT2A	-0.079	6.48096E-93
ZNF692	-0.08	2.43483E-91
AGER	-0.084	5.25566E-126
EFCAB6	-0.084	1.94044E-97
ZNF169	-0.084	1.45339E-113
GTF2IRD2	-0.085	2.47093E-109
LHX4	-0.086	1.72031E-137
TRIM52	-0.087	3.36803E-112
L3MBTL1	-0.091	4.77603E-110
GTF2IRD2B	-0.095	2.00736E-134
ZNF19	-0.112	1.27428E-144

Table S3: List of 156 significant MRs common across the majority of the prognostic cancers and are ranked based on fold change (FC) between activities in INV-H vs activities in INV-L samples across all the 10 cancers using Wilcoxon rank sum test.

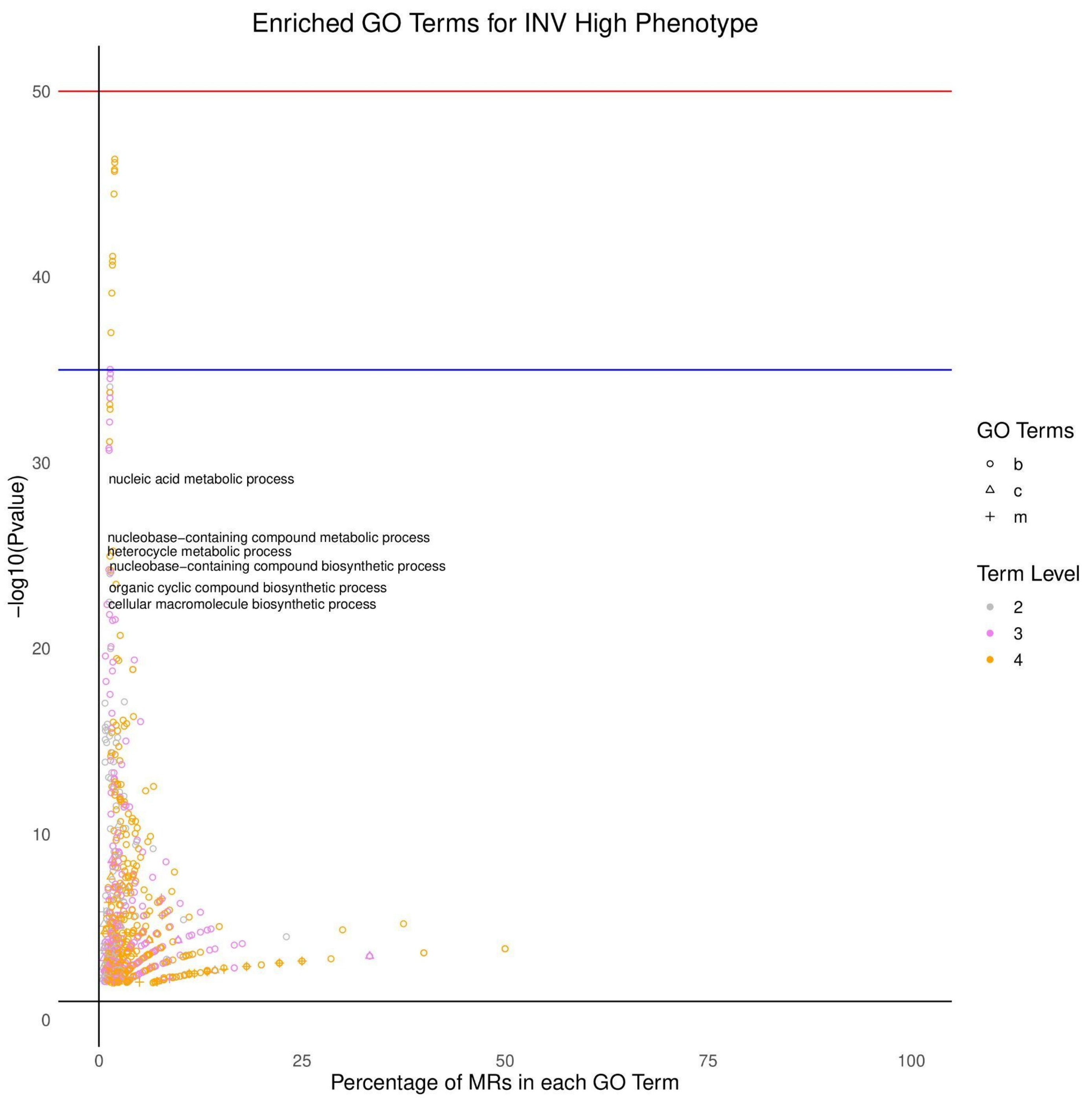

a) Most significant GO Terms associated with the common MRs specific to INV-H phenotype across 10 cancers. Here ‘b’ is for biological processes, ‘c’ is for cellular components, ‘m’ is for molecular functions.

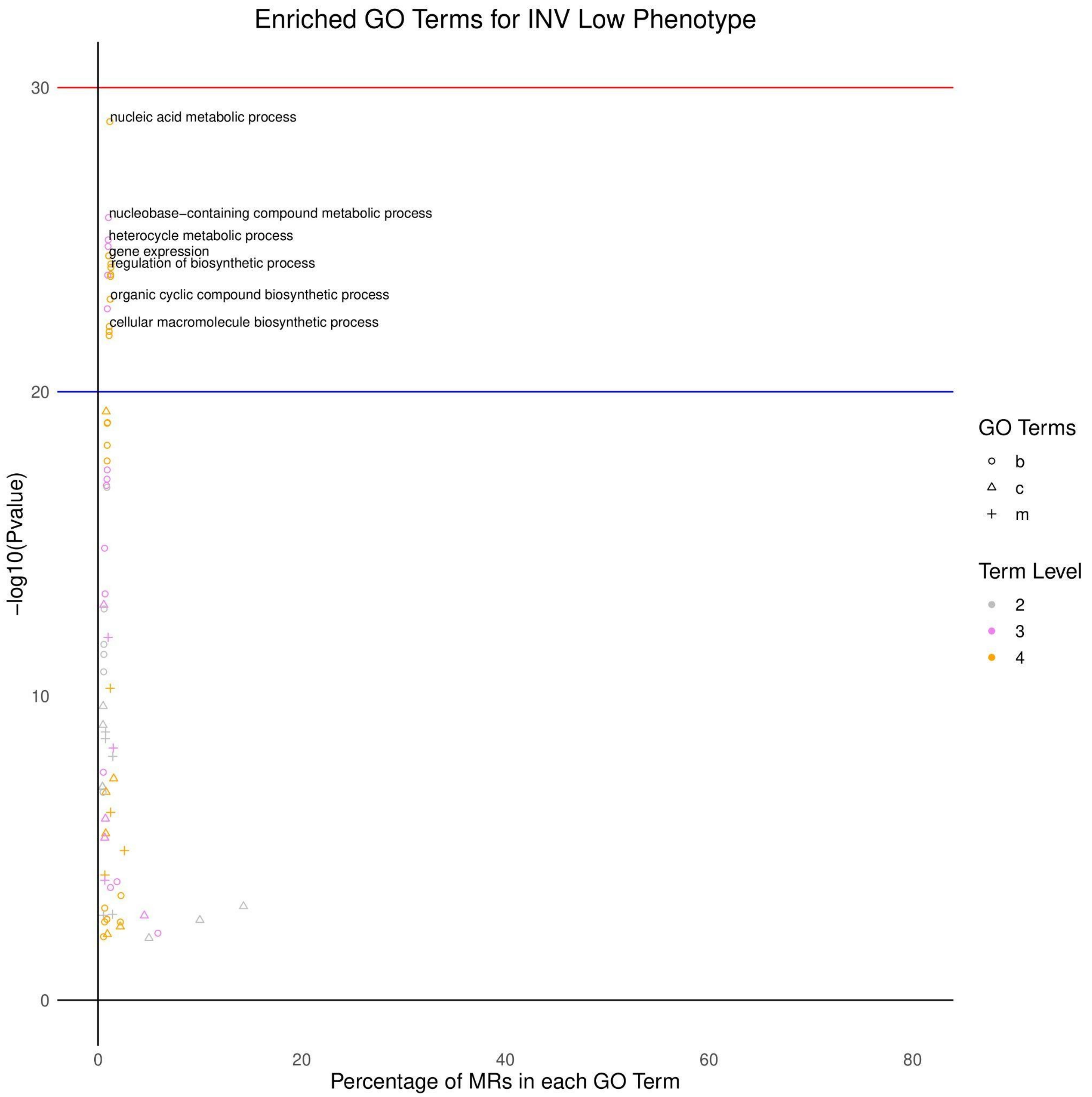

b) Most significant GO Terms associated with common MRs specific to INV-L phenotype across 10 cancers

Figure S5: GO Terms including Biological Processes (b), Cellular Components (c), Molecular Functions (m) which are significantly enriched when performing over-expression analysis of common MRs for INV-H and INV-L phenotype respectively.

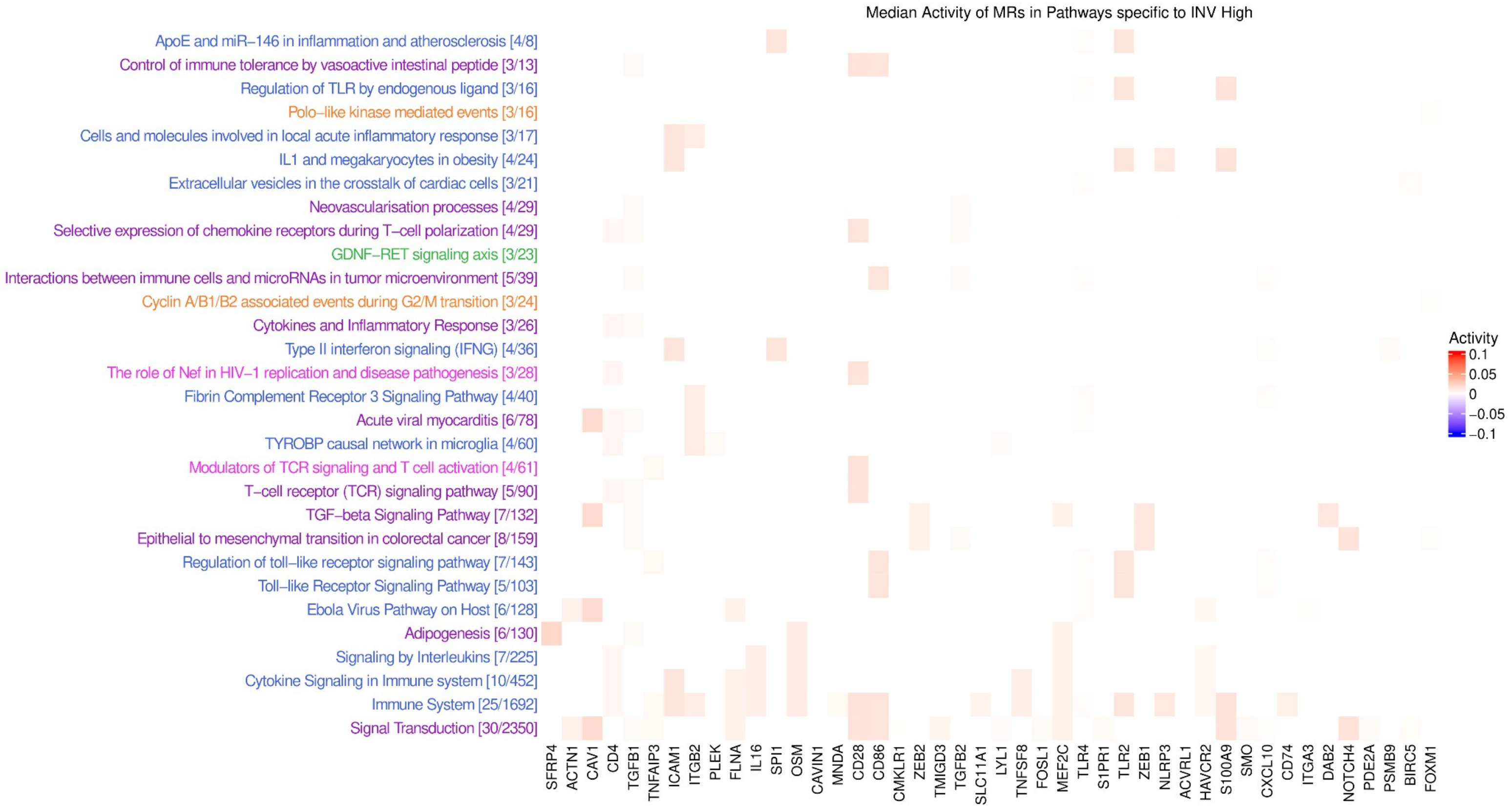

a) Heat plot showcasing the enriched pathways in which each of the top MRs are specific to INV High phenotype. The intensity represents the median activity of an MR across all the ten cancers of interest.

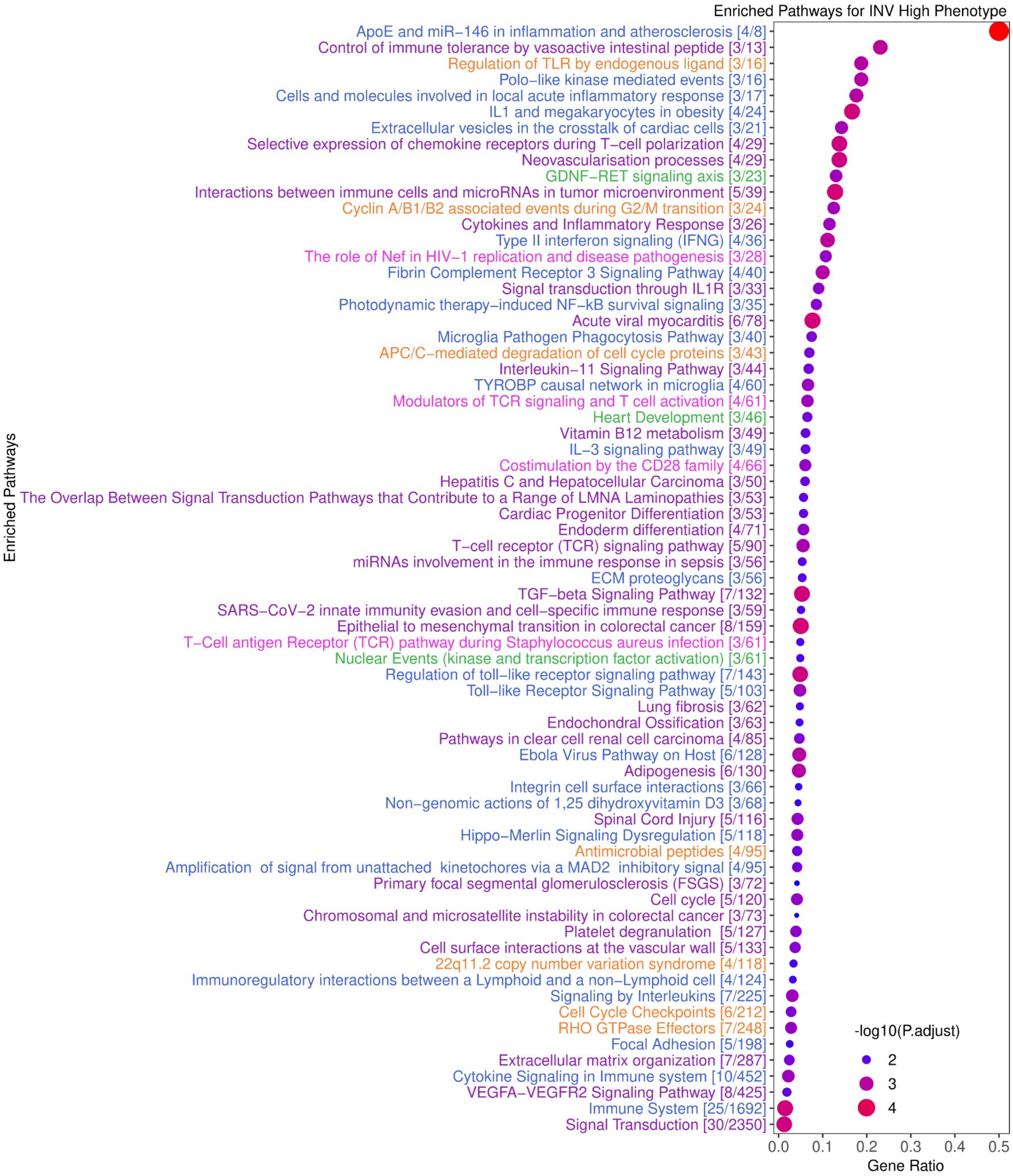

b) Dotplot showing the ratio between top MRs and the total number of genes in each enriched pathway, where the size of each dot is proportional to -$\log_{10}(\cdot)$ transformation on the adjusted p-values and the color of each dot increases from blue

to red as p-values decrease. The enriched pathways are colored grounded on the cluster to which each pathway belongs based on overlap between the top MRs in the pathways.

Figure S6: The top enriched pathways obtained by over-expression analysis for the top MRs peculiar to INV High phenotype are highlighted here. The pathways are clustered and color-coded according to their respective clusters

MRs	P-value	Mean1	Mean2	FC Mean	P-adjust
ZNF789	2.54E-207	-0.049	0.049	-0.098	6.6E-206
ANKRD49	2.33E-168	-0.049	0.045	-0.094	3.63E-167
BOLA1	1.77E-131	-0.043	0.042	-0.085	1.25E-130
TRIM52	1.39E-127	-0.039	0.045	-0.084	7.76E-127
ZNF692	2.97E-163	-0.039	0.041	-0.08	3.86E-162
ELP6	6.47E-118	-0.039	0.038	-0.077	2.52E-117
KAT2A	1.87E-146	-0.038	0.039	-0.077	2.08E-145
ALKBH4	8.46E-123	-0.041	0.035	-0.076	3.88E-122
GLI4	8.34E-108	-0.037	0.038	-0.075	2.89E-107
CDK5RAP3	4E-120	-0.035	0.04	-0.075	1.69E-119
PAXBP1	7.98E-126	-0.037	0.038	-0.075	4.29E-125
ZNF354B	2.15E-138	-0.04	0.035	-0.075	1.77E-137
HSF4	1.37E-139	-0.036	0.039	-0.075	1.19E-138
HDAC10	1.1E-100	-0.038	0.035	-0.073	3.35E-100
ZNF19	4.93E-112	-0.038	0.035	-0.073	1.79E-111
RECQL5	7.96E-123	-0.038	0.033	-0.072	3.76E-122
GTF2IRD2	2.31E-97	-0.034	0.036	-0.071	6.69E-97
PTGES2	3.77E-93	-0.035	0.033	-0.069	1.03E-92
ZC3H8	2.12E-85	-0.033	0.034	-0.067	5.08E-85
ZNF169	5.03E-106	-0.033	0.034	-0.067	1.63E-105
PIDD1	1.65E-87	-0.032	0.034	-0.066	4.08E-87
CDK11A	7.75E-79	-0.036	0.026	-0.062	1.63E-78

ZNF23	1.2E-72	-0.033	0.028	-0.06	2.29E-72
MXD3	3.28E-73	-0.03	0.03	-0.06	6.4E-73
ZNF133	5.34E-78	-0.033	0.026	-0.059	1.1E-77
ZNF337	2E-83	-0.027	0.032	-0.059	4.67E-83
PABPC1L	1.19E-86	-0.028	0.029	-0.058	2.9E-86
ZRANB2	3.28E-88	-0.032	0.027	-0.058	8.25E-88
GTF2IRD2B	1.48E-62	-0.027	0.029	-0.056	2.46E-62
APBB3	2.24E-68	-0.025	0.028	-0.054	3.97E-68
SARNP	5.76E-71	-0.029	0.025	-0.054	1.06E-70
MED31	1.33E-60	-0.031	0.022	-0.053	2.14E-60
PNN	3.37E-66	-0.03	0.023	-0.053	5.78E-66
MRPL12	2.64E-69	-0.029	0.024	-0.053	4.74E-69
CENPT	2.34E-82	-0.028	0.025	-0.053	5.22E-82
LHX4	5.56E-74	-0.025	0.027	-0.052	1.1E-73
MED11	3.34E-63	-0.027	0.024	-0.051	5.6E-63
AGER	6.81E-81	-0.028	0.023	-0.051	1.48E-80
MZF1	4.32E-52	-0.024	0.024	-0.049	6.35E-52
RBM39	4.53E-53	-0.024	0.024	-0.048	6.73E-53
L3MBTL1	1.4E-54	-0.023	0.024	-0.047	2.13E-54
TSSK4	6.27E-62	-0.023	0.023	-0.047	1.02E-61
CCNL2	5.64E-73	-0.023	0.025	-0.047	1.09E-72
FBXL15	3.92E-53	-0.028	0.018	-0.046	5.88E-53
SAP18	2.81E-53	-0.025	0.021	-0.046	4.25E-53
GATAD1	8.93E-38	-0.019	0.026	-0.044	1.22E-37
DMTF1	6.34E-56	-0.02	0.023	-0.043	9.89E-56
LPIN3	1.68E-33	-0.023	0.018	-0.041	2.16E-33
SLIRP	2.42E-42	-0.021	0.019	-0.04	3.37E-42
DDX39B	1.18E-50	-0.019	0.021	-0.04	1.73E-50

EFCAB6	8.01E-31	-0.019	0.019	-0.038	1.02E-30
MSRB2	5.36E-36	-0.02	0.018	-0.038	7.27E-36
ZNF276	4.99E-35	-0.02	0.017	-0.037	6.65E-35
ZNF688	2.12E-29	-0.019	0.017	-0.036	2.62E-29
STK36	1.16E-29	-0.014	0.022	-0.036	1.47E-29
CARF	1.7E-29	-0.013	0.022	-0.035	2.12E-29
TAF1C	1.4E-33	-0.019	0.015	-0.035	1.81E-33
ZNF862	5.17E-25	-0.013	0.02	-0.033	6.12E-25
CDC45	2.29E-22	-0.018	0.014	-0.032	2.61E-22
ZNF785	3.01E-24	-0.017	0.015	-0.032	3.51E-24
TCEA3	2.04E-19	-0.014	0.016	-0.03	2.27E-19
DEPDC1	2.75E-23	-0.017	0.012	-0.029	3.15E-23
PLK1	7.6E-19	-0.015	0.012	-0.027	8.4E-19
CENPF	2.19E-15	-0.013	0.012	-0.025	2.39E-15
DDX17	4.27E-17	-0.009	0.016	-0.025	4.7E-17
CCNA2	3.28E-15	-0.016	0.008	-0.024	3.56E-15
AURKB	7.22E-08	-0.012	0.005	-0.017	7.67E-08
DLGAP5	3.4E-07	-0.01	0.006	-0.016	3.53E-07
BIRC5	1.71E-07	-0.011	0.005	-0.015	1.79E-07
TAF1D	0.000326	-0.012	0	-0.012	0.00033
FOXM1	9.07E-05	-0.006	0.005	-0.011	9.25E-05
TOP2A	6.81E-05	-0.005	0.007	-0.011	7.04E-05
FANK1	0.00482	-0.007	0.001	-0.008	0.00485
ATP8B1	0.857	0.002	0.001	0.001	0.857
PSMB9	7.72E-05	0.006	-0.008	0.014	7.92E-05
SMO	9.82E-08	0.005	-0.011	0.016	1.03E-07
APBB1	7.43E-09	0.009	-0.009	0.017	7.94E-09
SOX18	1.41E-11	0.007	-0.01	0.017	1.52E-11

EOMES	8E-24	0.013	-0.014	0.027	9.25E-24
LYL1	1.17E-21	0.016	-0.012	0.028	1.32E-21
HOPX	6.6E-21	0.014	-0.017	0.031	7.41E-21
MECOM	5.03E-28	0.017	-0.017	0.034	6.04E-28
POU2AF1	2.7E-24	0.019	-0.016	0.035	3.17E-24
FOSL1	1.37E-28	0.012	-0.024	0.037	1.67E-28
SOX17	3.73E-29	0.019	-0.019	0.038	4.58E-29
HAND2	6.37E-28	0.019	-0.021	0.039	7.58E-28
PYHIN1	2.1E-28	0.018	-0.021	0.039	2.55E-28
CD3D	6.34E-30	0.018	-0.021	0.039	8.04E-30
CXCL10	5.97E-34	0.017	-0.023	0.04	7.83E-34
HMGA2	1.17E-35	0.016	-0.024	0.04	1.57E-35
PDE2A	6.84E-35	0.02	-0.023	0.043	9.04E-35
NFAM1	8.65E-56	0.021	-0.022	0.043	1.34E-55
S100A9	3.73E-41	0.018	-0.029	0.047	5.16E-41
APOE	2.24E-49	0.02	-0.027	0.047	3.2E-49
ERG	2.8E-57	0.024	-0.023	0.048	4.41E-57
MYOCD	1.05E-48	0.027	-0.022	0.049	1.49E-48
NCF1	1.37E-50	0.026	-0.024	0.049	1.97E-50
CDH13	1.79E-62	0.027	-0.022	0.049	2.94E-62
ITGA3	1.42E-48	0.025	-0.027	0.051	2E-48
SLC11A1	7.88E-67	0.021	-0.03	0.051	1.38E-66
NOTCH3	1.09E-59	0.029	-0.026	0.055	1.73E-59
RHOH	1.77E-66	0.027	-0.028	0.055	3.06E-66
TGFB1	7.61E-72	0.028	-0.031	0.058	1.41E-71
TLR4	1.23E-89	0.027	-0.031	0.058	3.14E-89
ZEB1	5.17E-70	0.032	-0.026	0.059	9.38E-70
CD28	5.64E-65	0.03	-0.03	0.06	9.57E-65

CD74	6.72E-72	0.034	-0.028	0.062	1.26E-71
TNFAIP3	2.79E-83	0.028	-0.034	0.062	6.39E-83
MEIS3	6.04E-84	0.029	-0.033	0.062	1.43E-83
TNFSF8	2.58E-74	0.031	-0.032	0.063	5.16E-74
IL6	3.06E-77	0.027	-0.036	0.063	6.19E-77
HCK	1.05E-78	0.029	-0.034	0.063	2.18E-78
FLNA	1.18E-79	0.031	-0.031	0.063	2.53E-79
OSM	1.52E-97	0.029	-0.036	0.064	4.46E-97
ACTN1	2.08E-91	0.033	-0.034	0.067	5.51E-91
TMIGD3	1.55E-90	0.036	-0.032	0.068	4.04E-90
S1PR1	1.51E-97	0.034	-0.034	0.068	4.46E-97
KANK2	5.91E-83	0.036	-0.033	0.069	1.34E-82
NOTCH4	1.73E-91	0.031	-0.038	0.069	4.65E-91
LZTS1	3.7E-94	0.035	-0.034	0.069	1.03E-93
MNDA	5.97E-103	0.034	-0.038	0.072	1.86E-102
NLRP3	6.95E-120	0.035	-0.037	0.072	2.78E-119
TLR2	1E-81	0.031	-0.042	0.073	2.2E-81
IKZF1	4.68E-97	0.037	-0.036	0.073	1.33E-96
ITGB2	4.21E-106	0.036	-0.036	0.073	1.4E-105
MEF2C	1.68E-105	0.036	-0.038	0.074	5.34E-105
CD86	8.13E-107	0.035	-0.039	0.074	2.76E-106
ICAM1	3.62E-110	0.037	-0.038	0.075	1.28E-109
PLEK	1.72E-114	0.039	-0.037	0.075	6.56E-114
HAVCR2	5E-128	0.037	-0.038	0.075	2.89E-127
TGFB1I1	2.82E-132	0.036	-0.041	0.077	2.1E-131
DKK3	6.95E-129	0.039	-0.038	0.078	4.34E-128
TGFB2	2.68E-112	0.038	-0.041	0.079	9.94E-112
VGLL3	2.32E-122	0.038	-0.04	0.079	1.03E-121

TFEC	1.08E-128	0.038	-0.041	0.079	6.48E-128
CAVIN1	1.13E-123	0.038	-0.042	0.08	5.49E-123
LILRB4	5.24E-129	0.039	-0.041	0.08	3.41E-128
IL16	2.25E-124	0.04	-0.041	0.081	1.13E-123
DAB2	1.97E-124	0.039	-0.042	0.081	1.02E-123
BNC2	1.53E-129	0.045	-0.037	0.082	1.03E-128
CAV1	2.9E-120	0.039	-0.044	0.083	1.26E-119
ACVRL1	6.99E-136	0.038	-0.045	0.083	5.45E-135
CYR61	4.7E-120	0.041	-0.044	0.085	1.93E-119
SPI1	4.82E-141	0.041	-0.044	0.085	4.7E-140
FLI1	4.29E-146	0.042	-0.043	0.085	4.46E-145
CMKLR1	7.5E-150	0.046	-0.039	0.085	9E-149
CD4	8.85E-140	0.044	-0.043	0.087	8.12E-139
MSC	1.27E-168	0.044	-0.048	0.093	2.2E-167
GREM1	2.08E-170	0.049	-0.044	0.093	4.06E-169
FOXC2	1.15E-165	0.045	-0.048	0.094	1.63E-164
ZEB2	7.09E-181	0.046	-0.05	0.095	1.58E-179
ZNF469	2.98E-208	0.051	-0.053	0.104	9.3E-207
SFRP4	6.25E-233	0.053	-0.057	0.11	3.25E-231
PRRX1	1.53E-226	0.055	-0.06	0.115	5.98E-225
SULF1	5.16E-242	0.061	-0.056	0.117	4.03E-240
DCN	6.08E-251	0.055	-0.064	0.119	9.48E-249

Table S4: List of 156 MRs specific to INV-L and INV-H phenotype and their activity profile for the set of 22 INV Neutral cancers. The significance (155 out of 156) of difference in activities in INV-H vs INV-L cancer samples is highlighted using Wilcoxon rank-sum test.

**MRs**	**P-value**	**Mean1**	**Mean2**	**FC Mean**	**P-adjust**
ZC3H8	4.28E-97	-0.036	0.033	-0.069	2.3E-96

MZF1	7.55E-92	-0.036	0.031	-0.067	3.37E-91
AGER	1.21E-110	-0.033	0.031	-0.064	1.45E-109
CARF	1.77E-71	-0.031	0.03	-0.062	4.93E-71
ZNF789	6.8E-95	-0.03	0.028	-0.058	3.31E-94
ZRANB2	3.59E-80	-0.03	0.027	-0.057	1.3E-79
APBB3	8.18E-102	-0.031	0.027	-0.057	5.12E-101
ZNF692	9.6E-103	-0.03	0.027	-0.057	6.81E-102
PAXBP1	1.1E-75	-0.031	0.025	-0.056	3.51E-75
CDK5RAP3	8.72E-103	-0.029	0.026	-0.055	6.48E-102
ZNF19	4.07E-71	-0.027	0.026	-0.053	1.11E-70
RECQL5	7.52E-78	-0.027	0.024	-0.051	2.61E-77
TRIM52	1.04E-71	-0.027	0.023	-0.05	2.94E-71
LHX4	1.14E-83	-0.026	0.023	-0.05	4.45E-83
L3MBTL1	8.4E-50	-0.025	0.024	-0.049	1.6E-49
ZNF169	2.12E-54	-0.024	0.025	-0.049	4.54E-54
GTF2IRD2	2.22E-82	-0.024	0.024	-0.048	8.44E-82
STK36	2.23E-73	-0.024	0.022	-0.047	6.45E-73
ZNF276	6.22E-71	-0.025	0.021	-0.046	1.67E-70
HSF4	6.59E-64	-0.024	0.021	-0.045	1.51E-63
CENPT	4.48E-65	-0.024	0.021	-0.045	1.06E-64
LPIN3	1.2E-69	-0.025	0.021	-0.045	3.01E-69
BOLA1	1.04E-31	-0.024	0.02	-0.044	1.52E-31
GLI4	1.06E-48	-0.024	0.02	-0.044	1.96E-48
ZNF785	3.82E-53	-0.021	0.022	-0.044	7.84E-53
CCNL2	3E-68	-0.024	0.02	-0.044	7.3E-68
PTGES2	2.26E-47	-0.024	0.019	-0.043	4.06E-47
MXD3	7.95E-36	-0.023	0.02	-0.042	1.2E-35
PNN	3.66E-51	-0.023	0.019	-0.042	7.05E-51

EFCAB6	3.53E-36	-0.022	0.019	-0.041	5.4E-36
ANKRD49	8.92E-37	-0.022	0.018	-0.04	1.41E-36
TSSK4	3.44E-70	-0.02	0.019	-0.039	8.93E-70
SAP18	6.59E-42	-0.021	0.017	-0.038	1.09E-41
TAF1C	4.36E-44	-0.021	0.017	-0.038	7.56E-44
GTF2IRD2B	1.23E-40	-0.02	0.017	-0.037	2.02E-40
RBM39	1.06E-47	-0.02	0.018	-0.037	1.92E-47
TCEA3	2.58E-29	-0.02	0.016	-0.036	3.7E-29
FANK1	1.75E-22	-0.018	0.013	-0.032	2.29E-22
ELP6	8.46E-25	-0.017	0.015	-0.032	1.16E-24
ZNF337	2.53E-36	-0.017	0.014	-0.032	3.91E-36
ZNF23	5.63E-22	-0.019	0.012	-0.031	7.32E-22
ALKBH4	2.63E-25	-0.017	0.014	-0.031	3.63E-25
DDX39B	2.03E-38	-0.017	0.014	-0.031	3.27E-38
DDX17	1.12E-30	-0.017	0.013	-0.03	1.61E-30
PIDD1	7.47E-19	-0.015	0.014	-0.029	9.55E-19
HDAC10	7.88E-23	-0.016	0.013	-0.029	1.04E-22
TOP2A	9.3E-18	-0.016	0.012	-0.028	1.18E-17
CDK11A	8E-14	-0.015	0.011	-0.027	9.52E-14
GATAD1	6.96E-14	-0.014	0.011	-0.025	8.35E-14
MECOM	8.1E-15	-0.014	0.012	-0.025	9.87E-15
ZNF354B	2.28E-24	-0.014	0.011	-0.025	3.1E-24
ZNF688	3.11E-16	-0.013	0.01	-0.024	3.84E-16
SARNP	1.57E-16	-0.013	0.011	-0.024	1.96E-16
MRPL12	2.6E-13	-0.014	0.01	-0.023	3.07E-13
DMTF1	2.14E-19	-0.013	0.01	-0.023	2.76E-19
SLIRP	2.93E-15	-0.012	0.01	-0.022	3.6E-15
CDC45	1.19E-09	-0.012	0.008	-0.021	1.36E-09

TAF1D	8.43E-09	-0.013	0.008	-0.02	9.53E-09
BIRC5	3.37E-11	-0.012	0.008	-0.02	3.92E-11
MED11	4.63E-14	-0.011	0.009	-0.02	5.6E-14
FBXL15	1.6E-17	-0.012	0.008	-0.02	2.01E-17
AURKB	3.95E-10	-0.011	0.008	-0.019	4.57E-10
ZNF133	9.06E-07	-0.012	0.006	-0.018	1E-06
FOXM1	5.25E-08	-0.012	0.006	-0.018	5.9E-08
CENPF	1.31E-06	-0.011	0.005	-0.016	1.44E-06
DLGAP5	8.03E-06	-0.009	0.005	-0.014	8.7E-06
PABPC1L	1.37E-12	-0.008	0.005	-0.013	1.6E-12
PLK1	5.83E-05	-0.008	0.004	-0.012	6.18E-05
MSRB2	0.000195	-0.007	0.004	-0.011	0.000206
DEPDC1	0.0044	-0.007	0.003	-0.01	0.00458
MED31	0.00446	-0.005	0.002	-0.007	0.0046
CCNA2	0.305	-0.005	0.001	-0.005	0.309
KAT2A	0.000232	-0.003	0.002	-0.005	0.000243
ZNF862	0.326	-0.001	0.001	-0.003	0.328
CYR61	1	0	0	0	1
ATP8B1	0.0243	0.002	-0.006	0.007	0.025
APBB1	0.0273	0.002	-0.006	0.008	0.0278
ITGA3	3.67E-09	0.004	-0.007	0.011	4.18E-09
POU2AF1	8.84E-06	0.006	-0.008	0.013	9.51E-06
MYOCD	4.42E-06	0.005	-0.008	0.013	4.82E-06
LYL1	1.04E-05	-0.018	-0.032	0.014	1.11E-05
HMGA2	1.94E-07	0.006	-0.011	0.016	2.16E-07
CDH13	6.31E-23	0.009	-0.009	0.019	8.41E-23
SOX18	5.58E-27	0.015	-0.02	0.035	7.84E-27
PSMB9	7.76E-28	0.016	-0.02	0.036	1.1E-27

SOX17	3.16E-23	0.016	-0.021	0.037	4.25E-23
PDE2A	8.88E-26	0.016	-0.022	0.038	1.24E-25
CD3D	1.21E-36	0.017	-0.021	0.038	1.88E-36
CD28	1.51E-35	0.017	-0.023	0.04	2.26E-35
HOPX	3.74E-39	0.018	-0.023	0.041	6.08E-39
ZEB2	1.93E-34	0.019	-0.026	0.044	2.87E-34
S1PR1	5.85E-37	0.019	-0.025	0.044	9.31E-37
TNFSF8	4.04E-42	0.019	-0.025	0.044	6.78E-42
DKK3	1.31E-33	0.018	-0.027	0.045	1.93E-33
S100A9	3.42E-52	0.02	-0.026	0.046	6.85E-52
TMIGD3	3.55E-54	0.02	-0.026	0.046	7.49E-54
EOMES	1.89E-42	0.022	-0.026	0.048	3.21E-42
MEF2C	1.28E-42	0.022	-0.026	0.048	2.2E-42
CD74	3.81E-47	0.022	-0.026	0.048	6.75E-47
APOE	4.6E-52	0.023	-0.026	0.049	9.09E-52
NCF1	3.48E-53	0.022	-0.027	0.049	7.23E-53
ZEB1	5.17E-48	0.022	-0.028	0.05	9.48E-48
PYHIN1	2.54E-51	0.022	-0.027	0.05	4.94E-51
NFAM1	1.07E-55	0.021	-0.029	0.05	2.35E-55
TGFB2	6.43E-49	0.023	-0.028	0.051	1.21E-48
TLR2	7E-53	0.022	-0.029	0.051	1.42E-52
HAND2	4.89E-46	0.024	-0.029	0.053	8.57E-46
TLR4	7.82E-55	0.024	-0.03	0.054	1.69E-54
TFEC	4.38E-77	0.025	-0.03	0.055	1.49E-76
SULF1	5.53E-66	0.027	-0.029	0.056	1.33E-65
CXCL10	2E-62	0.026	-0.032	0.057	4.52E-62
NOTCH3	1.33E-60	0.025	-0.032	0.058	2.95E-60
CAV1	7.38E-79	0.026	-0.034	0.06	2.62E-78

NOTCH4	5.38E-70	0.027	-0.034	0.061	1.38E-69
HCK	3.54E-81	0.028	-0.033	0.061	1.31E-80
ZNF469	2.72E-70	0.027	-0.036	0.063	7.2E-70
LILRB4	1.42E-75	0.029	-0.034	0.063	4.44E-75
MNDA	7.95E-85	0.03	-0.034	0.064	3.18E-84
SMO	6.98E-89	0.029	-0.035	0.064	2.86E-88
FLNA	2.24E-104	0.029	-0.035	0.065	1.84E-103
DAB2	2.76E-68	0.03	-0.036	0.066	6.83E-68
RHOH	3.34E-76	0.03	-0.036	0.066	1.11E-75
SFRP4	2.04E-89	0.031	-0.035	0.066	8.61E-89
VGLL3	7.81E-76	0.03	-0.037	0.067	2.54E-75
ACVRL1	1.38E-94	0.03	-0.037	0.067	6.52E-94
ERG	7.65E-102	0.032	-0.035	0.067	5.12E-101
ACTN1	2.84E-100	0.032	-0.037	0.069	1.7E-99
KANK2	8.63E-65	0.042	-0.028	0.07	2.01E-64
GREM1	7.7E-74	0.03	-0.04	0.07	2.27E-73
TGFB1	2.5E-75	0.023	-0.047	0.07	7.65E-75
IKZF1	1.35E-90	0.033	-0.037	0.07	5.87E-90
SLC11A1	3.35E-95	0.031	-0.039	0.07	1.68E-94
FOSL1	6.08E-96	0.032	-0.038	0.07	3.16E-95
CD86	2.23E-92	0.032	-0.04	0.071	1.02E-91
BNC2	4.16E-98	0.032	-0.04	0.072	2.32E-97
HAVCR2	2.11E-98	0.033	-0.039	0.072	1.22E-97
FOXC2	2.56E-75	0.035	-0.041	0.075	7.68E-75
PLEK	1.79E-107	0.035	-0.04	0.075	1.74E-106
CMKLR1	1.48E-114	0.035	-0.04	0.075	2.11E-113
SPI1	2.63E-104	0.034	-0.042	0.076	2.05E-103
ITGB2	3.68E-106	0.035	-0.042	0.076	3.38E-105

CD4	2.43E-110	0.036	-0.04	0.076	2.71E-109
PRRX1	1.77E-123	0.037	-0.041	0.079	3.94E-122
LZTS1	8.21E-102	0.038	-0.044	0.081	5.12E-101
IL16	6.06E-110	0.038	-0.043	0.081	6.31E-109
FLI1	1.32E-126	0.036	-0.044	0.081	3.43E-125
MEIS3	6.37E-117	0.037	-0.046	0.083	1.1E-115
DCN	2.47E-115	0.038	-0.046	0.084	3.85E-114
MSC	1.27E-131	0.039	-0.046	0.084	4.94E-130
NLRP3	4.18E-105	0.038	-0.047	0.085	3.62E-104
ICAM1	5.69E-127	0.04	-0.047	0.087	1.77E-125
TNFAIP3	2.9E-112	0.04	-0.048	0.088	3.78E-111
CAVIN1	2.53E-134	0.04	-0.048	0.088	1.32E-132
OSM	6.97E-122	0.042	-0.048	0.09	1.36E-120
TGFB1I1	5.74E-151	0.044	-0.054	0.097	8.95E-149
IL6	4.26E-148	0.047	-0.054	0.101	3.33E-146

Table S5: List of 156 MRs specific to INV-L and INV-H phenotype and their activity profile for the set of 8 PRECOG datasets. The significance (153 out of 156) of the difference in activities in INV-H vs INV-L cancer samples is highlighted using the Wilcoxon rank-sum test.

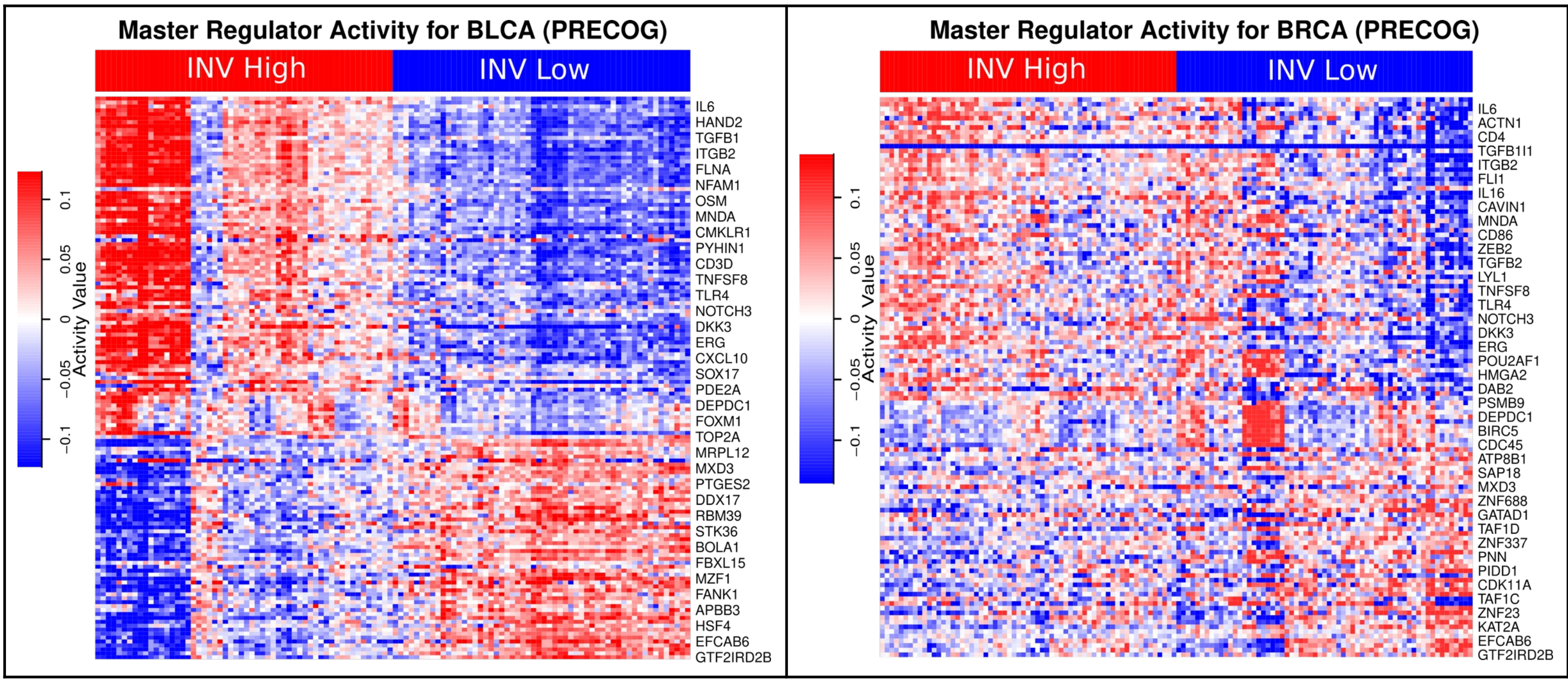
Master Regulator Activity for BLCA (PRECOG)
INV High
INV Low
Activity Value
0.1
0.05
0
−0.05
−0.1
IL6
HAND2
TGFB1
ITGB2
FLNA
NFAM1
OSM
MNDA
CMKLR1
PYHIN1
CD3D
TNFSF8
TLR4
NOTCH3
DKK3
ERG
CXCL10
SOX17
PDE2A
DEPDC1
FOXM1
TOP2A
MRPL12
MXD3
PTGES2
DDX17
RBM39
STK36
BOLA1
FBXL15
MZF1
FANK1
APBB3
HSF4
EFCAB6
GTF2IRD2B
Master Regulator Activity for BRCA (PRECOG)
INV High
INV Low
Activity Value
0.1
0.05
0
−0.05
−0.1
IL6
ACTN1
CD4
TGFB1I1
ITGB2
FLI1
IL16
CAVIN1
MNDA
CD86
ZEB2
TGFB2
LYL1
TNFSF8
TLR4
NOTCH3
DKK3
ERG
POU2AF1
HMGA2
DAB2
PSMB9
DEPDC1
BIRC5
CDC45
ATP8B1
SAP18
MXD3
ZNF688
GATAD1
TAF1D
ZNF337
PNN
PIDD1
CDK11A
TAF1C
ZNF23
KAT2A
EFCAB6
GTF2IRD2B

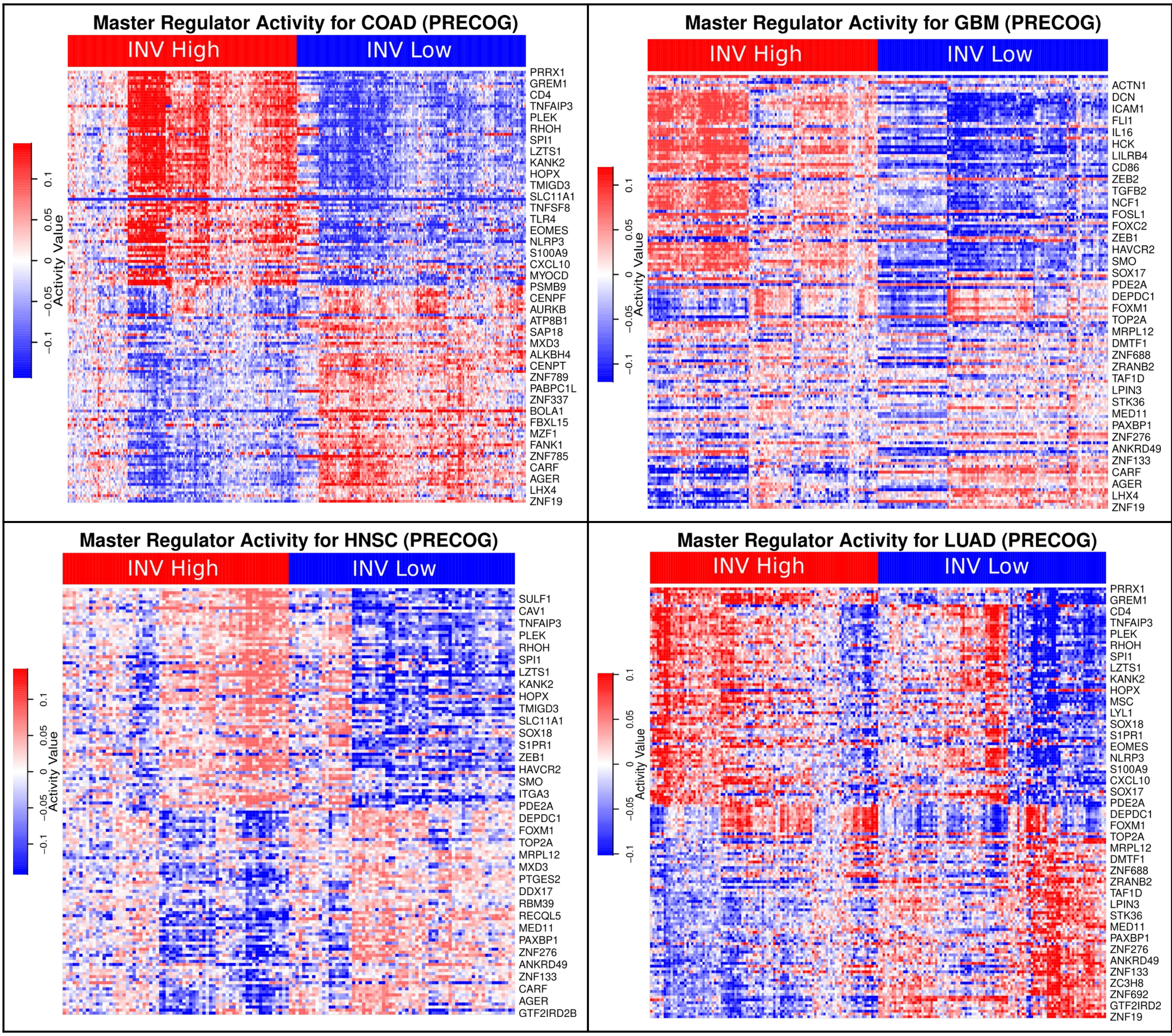

Master Regulator Activity for COAD (PRECOG)
INV High
INV Low
Activity Value
0.1
0.05
0
−0.05
−0.1
PRRX1
GREM1
CD4
TNFAIP3
PLEK
RHOH
SPI1
LZTS1
KANK2
HOPX
TMIGD3
SLC11A1
TNFSF8
TLR4
EOMES
NLRP3
S100A9
CXCL10
MYOCD
PSMB9
CENPF
AURKB
ATP8B1
SAP18
MXD3
ALKBH4
CENPT
ZNF789
PABPC1L
ZNF337
BOLA1
FBXL15
MZF1
FANK1
ZNF785
CARF
AGER
LHX4
ZNF19
Master Regulator Activity for GBM (PRECOG)
INV High
INV Low
Activity Value
0.1
0.05
0
−0.05
−0.1
ACTN1
DCN
ICAM1
FLI1
IL16
HCK
LILRB4
CD86
ZEB2
TGFB2
NCF1
FOSL1
FOXC2
ZEB1
HAVCR2
SMO
SOX17
PDE2A
DEPDC1
FOXM1
TOP2A
MRPL12
DMTF1
ZNF688
ZRANB2
TAF1D
LPIN3
STK36
MED11
PAXBP1
ZNF276
ANKRD49
ZNF133
CARF
AGER
LHX4
ZNF19
Master Regulator Activity for HNSC (PRECOG)
INV High
INV Low
Activity Value
0.1
0.05
0
−0.05
−0.1
SULF1
CAV1
TNFAIP3
PLEK
RHOH
SPI1
LZTS1
KANK2
HOPX
TMIGD3
SLC11A1
SOX18
S1PR1
ZEB1
HAVCR2
SMO
ITGA3
PDE2A
DEPDC1
FOXM1
TOP2A
MRPL12
MXD3
PTGES2
DDX17
RBM39
RECQL5
MED11
PAXBP1
ZNF276
ANKRD49
ZNF133
CARF
AGER
GTF2IRD2B
Master Regulator Activity for LUAD (PRECOG)
INV High
INV Low
Activity Value
0.1
0.05
0
−0.05
−0.1
PRRX1
GREM1
CD4
TNFAIP3
PLEK
RHOH
SPI1
LZTS1
KANK2
HOPX
MSC
LYL1
SOX18
S1PR1
EOMES
NLRP3
S100A9
CXCL10
SOX17
PDE2A
DEPDC1
FOXM1
TOP2A
MRPL12
DMTF1
ZNF688
ZRANB2
TAF1D
LPIN3
STK36
MED11
PAXBP1
ZNF276
ANKRD49
ZNF133
ZC3H8
ZNF692
GTF2IRD2
ZNF19

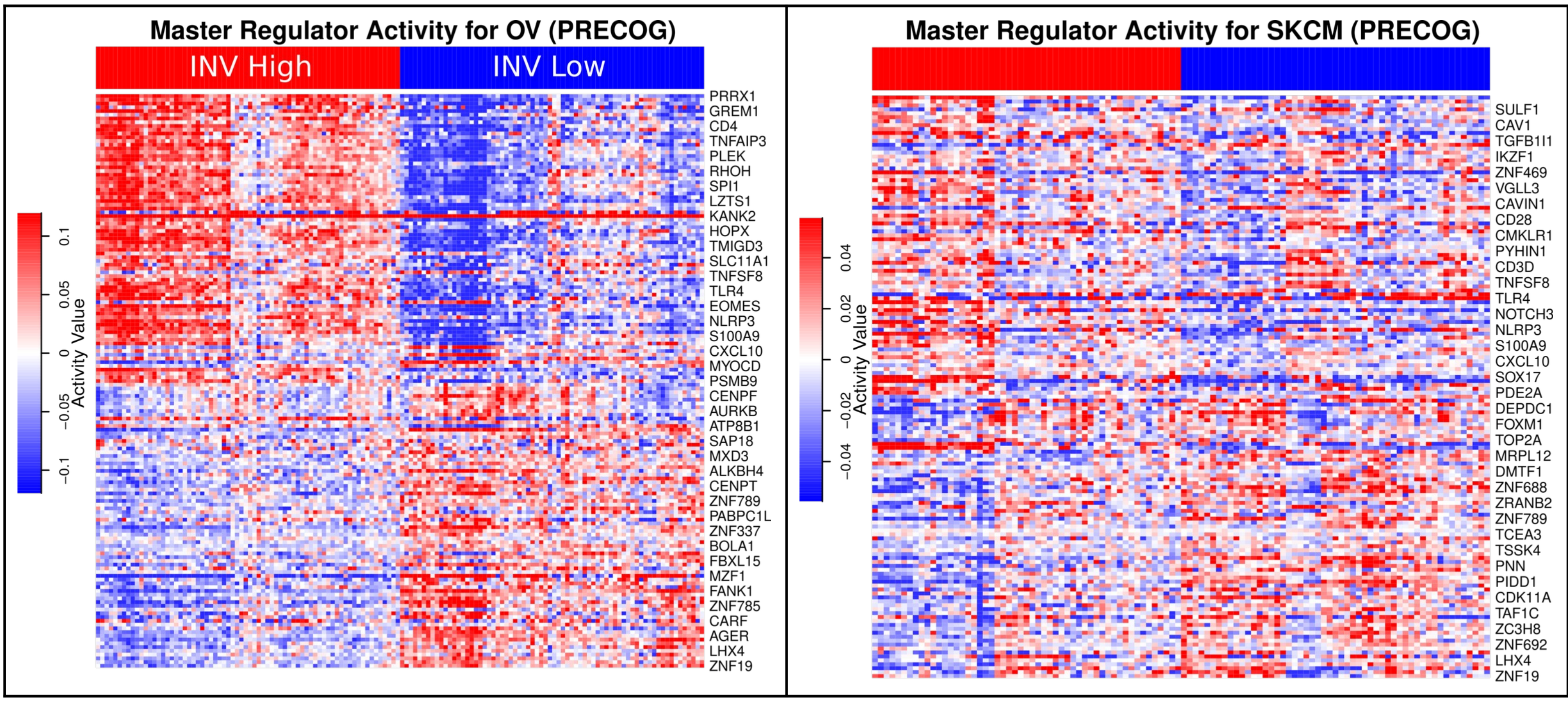

Figure S7: Heatmaps depicting the MR activity of the MRs specific to INV-H and INV-L phenotypes (based on the 10 prognostic cancers) and present in each of the 8 PRECOG validation datasets.

**Gene Name**	**Δ Mean (BLCA)**	**Padjust (BLCA)**	**Δ Mean (BRCA)**	**Padjust (BRCA)**	**Δ Mean (COAD)**	**Padjust (COAD)**	**Δ Mean (GBM)**	**Padjust (GBM)**
ACTN1	0.136	1.21E-16	0.048	0.000683	0.087	1.34E-20	0.087	4.01E-35
ACVRL1	0.11	3.72E-16	0.057	8.34E-06	0.002	0.253	0.112	1.28E-39
AGER	-0.108	2.3E-15	-0.025	0.000577	-0.111	7.93E-42	-0.023	0.00012
ALKBH4	-0.059	6E-10	-0.053	7.91E-06	-0.107	1.13E-38	0.058	9.88E-15
ANKRD49	-0.117	1.26E-14	-0.086	8.91E-07	-0.081	6.74E-34	0.049	3.42E-09
APBB1	0.124	5.55E-16	-0.007	0.876	0.106	1.09E-35	-0.171	1.28E-39
APBB3	-0.068	8.24E-09	-0.015	0.365	-0.097	1.36E-40	-0.069	1.13E-29
APOE	0.141	2.73E-15	0.004	1	0.122	1.56E-35	0	1
ATP8B1	-0.088	5.99E-14	-0.006	0.609	-0.012	0.00591	0.128	2.03E-39
AURKB	-0.004	0.454	-0.028	0.115	-0.066	3.25E-13	-0.013	0.0816
BIRC5	0.03	0.0136	-0.041	0.00129	-0.073	9.45E-24	-0.013	0.079
BNC2	0.113	1.12E-15	0.071	1.11E-07	0.14	1.4E-39	0.091	1.13E-29
BOLA1	-0.079	3.58E-11	-0.057	0.00166	-0.284	3.86E-45	0.067	3.93E-33
CARF	-0.085	4.74E-09	0.034	0.124	-0.036	1.84E-07	-0.186	2.45E-39

CAV1	0.156	1.58E-16	0.052	3.65E-05	0.15	2.38E-44	0.032	8.71E-10
CAVIN1	0.136	1.21E-16	0.05	6.94E-05	0.128	2.47E-41	0.162	2.2E-39
CCNA2	0.061	1.59E-08	-0.024	0.272	-0.039	2.8E-07	0.012	0.0358
CCNL2	-0.103	7.44E-15	0.011	0.272	-0.026	3.27E-06	-0.04	7.33E-13
CD28	0	1	-0.021	0.226	0.135	1.17E-34	0.058	2.42E-26
CD3D	0.085	6.58E-12	0.01	0.71	0.023	0.00478	0.098	3.14E-35
CD4	0.105	3.66E-16	0.066	1.11E-07	0.115	1.67E-35	0.104	9.26E-31
CD74	0.068	1.06E-09	0.083	1.79E-09	0.084	2.46E-23	0.087	8.96E-19
CD86	0.106	1.05E-15	0.016	0.36	0.137	6.48E-44	0.117	7.9E-38
CDC45	0.014	0.165	-0.075	9.78E-07	-0.106	2.03E-29	0.04	1.92E-09
CDH13	0.062	3.41E-06	0	1	0	1	0	1
CDK11A	-0.135	3.36E-09	-0.071	1.89E-05	-0.086	4.94E-19	0.125	2.1E-35
CDK5RAP3	-0.105	1.06E-14	-0.05	1.35E-05	-0.056	3.01E-23	-0.053	8.29E-29
CENPF	0.016	0.195	-0.042	0.0227	-0.019	0.00457	-0.027	0.000537
CENPT	-0.064	1.01E-12	-0.013	0.544	-0.11	3.63E-39	-0.036	1.49E-17
CMKLR1	0.131	5.92E-16	0.062	2.34E-05	0.093	1.38E-29	0.068	1.63E-20
CXCL10	0.103	8.34E-11	-0.033	0.0145	0.059	4.72E-12	0.152	1.28E-39
CYR61	0	1	0	1	0	1	0	1
DAB2	0.17	1.58E-16	0.008	1	0.055	4.09E-07	0.119	1.44E-35
DCN	0.131	3.67E-16	0.059	3.99E-07	0.148	2.55E-40	0.142	1.28E-39
DDX17	-0.083	3.15E-11	-0.007	0.449	0.007	0.566	-0.041	4.91E-20
DDX39B	-0.056	7.92E-08	-0.01	0.355	-0.038	4.25E-11	-0.011	0.00632
DEPDC1	0.025	0.00711	-0.042	0.00603	-0.068	1.53E-16	0.053	7.12E-16
DKK3	0.261	2.08E-14	0.039	4.55E-05	0.165	6.36E-46	-0.152	1.3E-21
DLGAP5	0.076	4.49E-11	-0.034	0.0215	-0.081	1.15E-22	-0.003	1
DMTF1	-0.103	2.29E-13	-0.015	0.153	0.011	0.0147	-0.011	0.0108
EFCAB6	-0.137	1.02E-12	0.007	1	-0.062	2.33E-15	0.046	1.72E-18
ELP6	-0.086	1.95E-13	0	1	-0.121	1.38E-42	0.058	1.34E-23

EOMES	0.075	1.37E-10	0	1	0.043	0.000156	0.08	7.7E-11
ERG	0.146	3.49E-16	0.07	6.62E-09	0.113	1.17E-38	0.077	1.12E-36
FANK1	-0.071	1.08E-08	0	1	-0.126	4.77E-43	0.144	2.13E-39
FBXL15	0.004	0.458	0.024	0.0458	-0.07	3.26E-35	0.014	0.00307
FLI1	0.123	1.21E-16	0.064	1.24E-05	0.153	1.12E-43	0.092	7.64E-31
FLNA	0.133	1.21E-16	0.02	0.0633	0.139	8.27E-45	0.075	1.56E-24
FOSL1	0.06	3.77E-07	0.029	0.165	0.062	1.63E-23	0.184	1.28E-39
FOXC2	0.096	3.34E-11	0.019	0.31	0.128	2.2E-13	0.151	1.28E-39
FOXM1	0.025	0.00119	-0.043	0.000683	-0.064	3.66E-16	-0.056	2.3E-10
GATAD1	-0.083	5.08E-10	0.055	0.0329	-0.111	8.9E-37	0.112	3.49E-37
GLI4	-0.073	5.63E-10	0	1	-0.054	1.79E-11	-0.096	4.55E-27
GREM1	0.105	4.72E-16	0.076	7.71E-05	0.195	1.02E-45	-0.068	5.35E-16
GTF2IRD2	0	1	0	1	-0.066	8.63E-25	-0.125	1.01E-31
GTF2IRD2B	-0.116	4.63E-14	-0.039	0.0016	-0.07	3.1E-21	-0.007	0.23
HAND2	0.122	1.79E-14	0.02	0.473	0.1	1.7E-35	0.024	0.000361
HAVCR2	0.113	3.51E-15	0	1	0.12	2.14E-37	0.112	7.8E-28
HCK	0.081	2.81E-13	0	1	0.112	2.42E-36	0.102	9.62E-33
HDAC10	-0.074	9.69E-12	0	1	-0.089	3.17E-32	0.058	1.04E-25
HMGA2	0.119	6.9E-15	0.054	0.0159	-0.133	7.28E-39	0.092	1.44E-35
HOPX	0.096	1.76E-09	0	1	0.117	5.8E-42	0.088	2.13E-33
HSF4	-0.049	1.29E-06	-0.008	0.812	-0.089	6.22E-32	-0.028	9.2E-07
ICAM1	0.137	1.21E-16	0.06	1.86E-05	0.16	3.86E-45	0.138	1.28E-39
IKZF1	0.153	2.62E-16	0.051	0.00081	0.124	3.64E-36	0.104	1.87E-23
IL16	0.114	2.53E-15	0.091	9.99E-10	0.121	1.07E-33	0.139	2.6E-39
IL6	0.068	9.99E-12	0.034	0.11	0.124	3.38E-33	0.174	2.12E-39
ITGA3	0.042	6.37E-05	0	1	0	1	0	1
ITGB2	0.117	3.72E-16	0.031	0.00695	0.154	2.25E-42	0.107	7.47E-32
KANK2	0.084	1.12E-15	0.043	0.00695	0.155	3.86E-45	0.179	1.28E-39

KAT2A	-0.104	3.98E-14	-0.092	1.37E-10	-0.105	5.58E-30	-0.088	2.43E-31
L3MBTL1	-0.081	3.34E-11	-0.007	0.545	-0.002	0.886	-0.166	2.96E-36
LHX4	-0.092	2.9E-13	0	1	-0.047	5.21E-20	-0.09	3.06E-22
LILRB4	0.11	3.6E-15	0.009	0.673	0.126	1.95E-38	0.033	2E-06
LPIN3	-0.126	1.85E-15	0	1	-0.053	3.66E-25	-0.058	9.68E-26
LYL1	0	1	0.068	1.87E-05	0	1	0.049	3.26E-16
LZTS1	0.108	3.98E-16	0.025	0.265	0.128	8.91E-33	0.158	1.28E-39
MECOM	-0.101	1.53E-13	0	1	-0.053	6.05E-14	0.11	1.3E-21
MED11	-0.092	1.58E-16	0	1	-0.04	2.35E-11	0.05	3.26E-25
MED31	0	1	-0.029	0.00752	-0.077	2.35E-31	0.095	5.79E-38
MEF2C	0.095	1.07E-15	0.058	6.67E-05	0.094	4.75E-32	0.047	0.00357
MEIS3	0.069	0.00126	0	1	0.151	2.1E-43	0.11	1.03E-25
MNDA	0.108	3.51E-15	0.005	1	0.105	2.74E-31	0.09	2.71E-33
MRPL12	-0.066	3.15E-11	-0.062	3.06E-06	-0.094	2.1E-30	0.076	1.12E-36
MSC	0.127	1.2E-15	0.044	0.00263	0.164	3.89E-44	0.063	2.1E-18
MSRB2	0.014	0.753	0	1	-0.08	2.37E-19	0.036	1.16E-05
MXD3	-0.097	1.32E-13	-0.028	0.609	-0.117	1.62E-27	-0.063	2.48E-16
MYOCD	0.019	0.0134	0	1	0.097	3.2E-35	-0.019	0.0251
MZF1	-0.151	1.21E-16	-0.005	0.383	-0.094	9.2E-39	-0.018	1.08E-05
NCF1	0.122	5.3E-15	-0.024	0.138	0.07	1.39E-16	0.091	9.99E-29
NFAM1	0.114	1.21E-16	0	1	0.023	0.0792	0.093	2.65E-33
NLRP3	0.11	3.02E-16	0.11	8.92E-10	0.256	1.49E-27	0.133	5.29E-37
NOTCH3	0.006	0.342	0.034	0.315	0.164	3.65E-43	0.1	3.53E-36
NOTCH4	0.01	0.3	0.066	6.99E-06	0.192	6.36E-46	0.063	4.91E-17
OSM	0.104	3.72E-13	0	1	0.152	7.46E-45	0.206	3.29E-39
PABPC1L	0	1	0	1	-0.045	3.1E-15	0.077	4.05E-31
PAXBP1	-0.105	4.3E-14	-0.096	1.79E-09	-0.061	5.35E-06	-0.055	2.05E-26
PDE2A	0.118	1.81E-09	0.075	1.13E-08	0.138	1.13E-38	-0.127	7.91E-39

PIDD1	-0.032	0.0218	-0.012	1	-0.028	0.00156	-0.002	0.858
PLEK	0.105	1.85E-15	0.037	0.00122	0.098	2.46E-32	0.126	4.34E-37
PLK1	0.027	0.00655	-0.05	7.91E-06	-0.05	1.78E-13	-0.016	0.027
PNN	-0.133	4.44E-16	-0.065	2.8E-05	-0.073	5.21E-27	-0.044	5.01E-17
POU2AF1	0.098	6.95E-13	-0.005	1	0.007	0.813	0	1
PRRX1	0.121	2.62E-16	0.047	0.000203	0.158	4.57E-45	0	1
PSMB9	0.064	6.17E-10	-0.032	0.000495	0.001	0.94	0.136	6.38E-37
PTGES2	-0.019	0.00826	-0.079	5.41E-05	-0.079	5.21E-30	-0.08	3.06E-23
PYHIN1	0.106	3.87E-14	-0.03	0.0243	0.084	9.8E-19	0.07	2.22E-26
RBM39	-0.106	1.99E-09	-0.009	0.421	-0.054	2.47E-18	-0.014	0.000601
RECQL5	-0.13	2.14E-14	-0.059	1.81E-07	-0.045	5.33E-15	-0.047	3.4E-25
RHOH	0.118	1.85E-15	0.023	0.203	0.078	4.14E-14	0.156	1.28E-39
S100A9	0.127	3.66E-16	-0.027	0.191	0.069	7.23E-21	0.111	1.18E-38
S1PR1	0.119	2.62E-16	0.062	9.94E-07	0.141	9.06E-43	-0.087	2.13E-33
SAP18	-0.085	4.01E-13	-0.03	0.008	-0.052	2.84E-18	-0.035	1.35E-07
SARNP	-0.079	1.53E-13	0	1	-0.115	3.33E-36	0.079	1.24E-29
SFRP4	0.119	3.71E-15	0.064	6.67E-05	0.154	7.61E-42	0	1
SLC11A1	0.081	7.36E-12	-0.02	0.225	0.165	3.89E-44	0.099	7.56E-33
SLIRP	-0.03	1.14E-05	-0.006	0.449	-0.11	1.24E-40	0.043	1.43E-15
SMO	0.116	1.02E-12	0.023	0.0633	0.122	2.99E-34	0.12	1.57E-37
SOX17	0.073	3.36E-09	0.019	0.177	0.106	5.26E-15	0.009	0.551
SOX18	0.093	4.57E-08	0	1	0.124	2.29E-38	-0.015	0.0512
SPI1	0.101	1.05E-15	0.031	0.0554	0.144	5.52E-43	0.114	3.35E-35
STK36	-0.074	1.14E-08	0	1	-0.053	3.58E-19	-0.018	0.000103
SULF1	0.1	7.31E-16	0.095	8.92E-10	0.109	1.27E-40	-0.016	0.0599
TAF1C	-0.065	9.54E-08	0.022	0.21	-0.024	0.000187	-0.084	1.23E-32
TAF1D	-0.112	2.53E-14	-0.057	0.000109	-0.071	1.69E-18	0.052	4.77E-24
TCEA3	-0.066	1.58E-06	0	1	-0.098	1.57E-24	0.025	0.027

TFEC	0	1	0.026	0.0166	0.125	7.23E-41	0.08	1.22E-32
TGFB1	0.068	4.17E-08	0	1	0.131	2.07E-37	0.133	1.12E-30
TGFB1I1	0.129	1.21E-16	0.027	0.017	0.169	2.15E-44	0.163	1.28E-39
TGFB2	0.135	2.47E-15	0.022	0.53	0.086	1.85E-21	0.062	1.15E-18
TLR2	0.062	1.26E-08	-0.007	0.726	0.086	4.07E-25	0.094	2.81E-35
TLR4	0.103	1.02E-12	0.079	2.19E-09	0.047	5.66E-07	0.071	1.85E-22
TMIGD3	0.137	1.49E-15	0	1	0.063	1.75E-16	0.068	8.36E-19
TNFAIP3	0.128	8.6E-16	0.052	0.0145	0.212	5.6E-38	0.134	2.66E-36
TNFSF8	0.114	3.27E-14	0.012	0.486	0.04	0.00376	0.111	2.03E-39
TOP2A	-0.042	3.88E-05	-0.021	0.0464	-0.126	9.26E-31	-0.021	0.00206
TRIM52	-0.114	2.08E-16	-0.01	0.545	-0.062	9.56E-23	-0.043	2.6E-18
TSSK4	0	1	0	1	-0.047	1.92E-18	-0.116	3.79E-28
VGLL3	0.014	0.04	0.087	2.34E-05	0.15	4.77E-43	0.027	0.0233
ZC3H8	-0.153	1.05E-15	0	1	-0.097	1.93E-26	-0.128	5.74E-37
ZEB1	0.12	2.62E-16	0.061	0.000143	0.126	2.72E-42	-0.068	1.7E-13
ZEB2	0.112	1.21E-16	0.063	3.73E-06	0.137	9.06E-43	-0.136	5.63E-38
ZNF133	-0.053	5.94E-06	-0.002	1	-0.032	0.0229	0.009	0.304
ZNF169	0	1	0	1	-0.167	2.02E-38	-0.013	0.0548
ZNF19	0	1	0	1	-0.135	1.67E-39	-0.049	4.19E-08
ZNF23	-0.104	2.94E-15	-0.044	0.0787	-0.053	1.5E-14	-0.015	0.23
ZNF276	-0.067	1.74E-07	-0.011	0.792	-0.017	9.14E-06	-0.088	2.84E-38
ZNF337	-0.078	1.84E-14	-0.002	0.609	-0.063	5.19E-20	0.012	0.00554
ZNF354B	0	1	0	1	-0.065	1.76E-28	0.029	9E-06
ZNF469	0.137	3.98E-16	0	1	0.175	6.48E-44	-0.042	1.38E-08
ZNF688	-0.087	2.9E-12	-0.03	0.0129	0.012	0.0452	0.014	0.000221
ZNF692	-0.089	3.51E-15	-0.031	1.35E-05	-0.094	1.94E-32	-0.043	5.16E-18
ZNF785	0	1	0	1	-0.229	1.63E-34	0	1
ZNF789	-0.117	1.81E-13	0	1	-0.068	2.44E-24	-0.101	1.13E-31

ZNF862	0	1	0.015	0.673	0.032	2.23E-05	0.002	0.55
ZRANB2	-0.084	1.64E-10	0	1	-0.085	4.04E-29	-0.03	0.000297
**Diff. Activated**		**136**		**68**		**146**		**136**
**Missing Mrs**		**13**		**36**		**3**		**8**

Table S6: Differentially activated MRs (out of 156 MRs) in the PRECOG cancer datasets for BLCA, BRCA, COAD and GBM cancer types.

**Gene Name**	**Δ Mean (HNSC)**	**Padjust (HNSC)**	**Mean (LUAD)**	**Padjust (LUAD)**	**Δ Mean (OV)**	**Padjust (OV)**	**FC Mean (SKCM)**	**Padjust (SKCM)**
ACTN1	0.075	2.58E-11	0.09	2.9E-21	0.043	9.6E-05	0.013	0.0115
ACVRL1	0.119	6.58E-17	0.034	0.000326	0.154	6.1E-20	0.042	3.51E-11
AGER	-0.062	4E-11	-0.094	4.08E-23	-0.065	2.19E-16	0.001	0.786
ALKBH4	-0.071	6.17E-11	0.032	3.8E-06	-0.086	4.21E-12	-0.03	1.88E-05
ANKRD49	-0.056	0.0012	-0.048	9.1E-07	-0.046	1.15E-05	-0.009	0.0586
APBB1	0.073	8.94E-10	-0.075	2.35E-16	0.113	3.9E-12	0.003	0.781
APBB3	-0.074	2.25E-13	-0.068	9.37E-24	-0.011	0.0565	-0.02	0.000377
APOE	0.033	0.0613	0.059	2.43E-09	0.049	8.31E-08	-0.002	1
ATP8B1	-0.059	1.06E-09	-0.037	4.4E-06	0.026	0.0393	0.042	0.000108
AURKB	-0.025	0.0234	0.037	2.63E-07	-0.031	1.98E-05	-0.01	0.0117
BIRC5	-0.055	9.7E-08	0.037	6E-06	-0.017	0.0253	0	0.868
BNC2	0.084	5.68E-10	0.066	7.64E-14	-0.018	0.113	-0.003	0.578
BOLA1	-0.037	0.00254	0.007	0.252	-0.057	4.41E-16	-0.015	0.0441
CARF	-0.072	3.74E-13	-0.092	1.37E-26	0.002	0.875	-0.02	0.002
CAV1	0.047	3.39E-06	0.003	0.687	0.054	2.69E-10	0.014	8.02E-05
CAVIN1	0.084	4E-11	0.029	0.000221	0.099	3.69E-19	0.009	0.027
CCNA2	-0.088	1.73E-10	0.032	1.52E-05	0.028	6.16E-05	-0.031	0.00109
CCNL2	-0.07	2.25E-13	-0.067	5.54E-22	-0.056	1.09E-14	-0.017	0.000804
CD28	0.049	5.04E-06	0.037	0.0044	-0.022	0.455	0.007	0.146
CD3D	-0.004	0.6	0.023	0.0258	0.088	2E-14	-0.005	0.081

CD4	0.066	3.22E-08	0.028	0.00784	0.114	4.59E-17	0.015	0.000495
CD74	0.006	1	0.009	0.523	0.037	0.00688	0.009	0.00917
CD86	0.06	1.83E-05	0.014	0.15	0.077	9.48E-13	-0.007	0.579
CDC45	-0.048	1.71E-06	0.041	6.92E-08	-0.032	0.000227	-0.007	0.0959
CDH13	0	1	0.089	7.84E-19	0	1	0.014	9.3E-05
CDK11A	-0.037	0.000115	-0.043	4.77E-07	-0.059	1.96E-07	-0.03	0.000222
CDK5RAP3	-0.026	0.0143	-0.068	5.96E-22	-0.069	1.88E-15	-0.016	3.24E-05
CENPF	-0.042	0.000197	0.045	5.85E-11	-0.049	5.38E-05	-0.04	2.96E-05
CENPT	-0.015	0.0252	-0.014	0.0342	-0.053	2.53E-09	-0.016	0.00656
CMKLR1	0.128	3.73E-17	0.064	1.99E-11	0.09	1.63E-17	0.011	0.00515
CXCL10	0.01	0.659	0.071	4.18E-17	0.029	0.00155	0.005	0.18
CYR61	0	1	0	1	0	1	0	1
DAB2	0.1	4.71E-12	0.025	0.0085	0.119	2.24E-20	-0.003	0.826
DCN	0.06	1.03E-09	-0.007	0.258	0.095	1.3E-14	0.048	1.05E-06
DDX17	-0.048	4.59E-06	-0.078	3.53E-19	-0.014	0.0337	0	1
DDX39B	-0.066	1.83E-08	-0.061	3.53E-19	0.009	0.0341	-0.02	2.56E-06
DEPDC1	-0.046	8.83E-05	0.024	0.000518	-0.029	0.000963	-0.007	0.406
DKK3	0.108	8.05E-17	0.003	0.939	0.065	2.4E-10	0.025	1.24E-06
DLGAP5	-0.025	0.00255	0.032	6.08E-06	-0.024	0.00226	-0.004	0.544
DMTF1	-0.024	0.00659	-0.062	1.16E-18	-0.026	0.011	-0.004	0.402
EFCAB6	-0.012	0.0628	-0.093	2.4E-18	-0.107	1.49E-21	-0.024	0.00118
ELP6	-0.012	0.0627	-0.011	0.45	-0.098	1.97E-14	0	0.776
EOMES	0	1	0.085	1.24E-16	0.149	5.49E-18	0	1
ERG	0	1	-0.008	0.129	0.133	1.33E-19	0.022	1.57E-05
FANK1	0.01	0.112	-0.102	3.79E-25	-0.13	1.49E-21	0	1
FBXL15	-0.019	0.0163	-0.054	1.27E-11	0.006	0.367	-0.025	2.93E-09
FLI1	0.069	1.13E-11	0.023	0.00784	0.109	3.15E-20	0.013	0.00157
FLNA	0.03	0.000158	0.034	2.2E-08	0.058	3.49E-14	-0.013	0.0255

FOSL1	0.014	0.286	0.069	2.47E-19	0.096	4.29E-15	0.002	0.544
FOXC2	0.055	4.32E-08	0.027	0.0253	0.106	1.97E-10	0.099	7.9E-08
FOXM1	-0.029	0.0219	0.066	8.81E-16	-0.012	0.0898	0.005	0.37
GATAD1	-0.03	0.0271	-0.069	3.52E-16	-0.058	1.92E-09	-0.011	0.00129
GLI4	-0.027	0.0145	-0.037	5.14E-06	-0.005	0.455	-0.024	5.19E-05
GREM1	0.057	6.02E-06	0.115	5.61E-26	0.147	2.16E-20	0.004	0.462
GTF2IRD2	0	1	-0.058	5.13E-18	-0.067	2.44E-10	0	1
GTF2IRD2B	-0.039	0.00828	0	1	-0.064	6.33E-16	-0.009	0.361
HAND2	0.277	8.47E-16	-0.064	1.36E-05	0.129	3.11E-19	0.019	0.00792
HAVCR2	0.072	3.49E-06	0.037	6.21E-05	0.114	2.39E-18	0.002	0.826
HCK	0.019	0.6	0.033	0.000835	0.095	4.56E-17	0.005	0.578
HDAC10	-0.058	8.03E-07	-0.049	2.28E-11	-0.027	0.0147	-0.017	0.00674
HMGA2	0.025	0.00417	0.101	1.69E-20	-0.066	1.81E-10	0.005	0.544
HOPX	-0.032	0.0123	-0.1	6.68E-23	0.111	2.32E-13	0.042	1E-09
HSF4	-0.039	3.22E-06	-0.069	1.7E-20	-0.03	1.36E-05	-0.016	0.000401
ICAM1	0.072	9.28E-09	0.035	8.97E-05	0.097	1.48E-12	-0.004	0.638
IKZF1	0.03	4.62E-05	0.042	8.69E-05	0.063	4.63E-11	0.005	0.162
IL16	0.076	4.3E-13	0.012	0.676	0.117	4.07E-11	0.018	0.0478
IL6	0.114	5.39E-18	0.125	4.53E-23	0.156	2.16E-20	0.024	3.76E-05
ITGA3	0.054	1.85E-08	0.014	0.0125	0	1	0.001	0.648
ITGB2	0.05	5.42E-05	0.034	0.000784	0.102	4.38E-18	-0.007	0.22
KANK2	0.068	4.03E-10	-0.038	0.000287	0	1	0.002	0.214
KAT2A	-0.078	7.98E-10	-0.034	4.67E-06	-0.032	0.00685	-0.008	0.0336
L3MBTL1	0	1	-0.062	2.68E-13	-0.064	1.04E-15	-0.003	0.628
LHX4	-0.06	4.55E-09	-0.064	6.34E-17	-0.029	5.63E-07	-0.004	0.304
LILRB4	0.186	7.3E-14	0.028	0.00077	0.074	7.99E-13	0.004	0.37
LPIN3	-0.041	0.00476	-0.057	3.25E-16	-0.007	0.601	0.002	0.211

LYL1	0	1	-0.01	0.38	0.018	0.34	0	1
LZTS1	0.04	0.0189	0.044	1.04E-09	0.132	1.49E-21	-0.032	2.06E-05
MECOM	0.002	0.992	-0.093	1.91E-19	-0.132	2.6E-19	0.06	8.36E-15
MED11	-0.035	0.000785	-0.026	0.00144	-0.033	3E-06	-0.05	3.81E-11
MED31	0	1	-0.003	0.735	-0.026	3.58E-06	-0.029	0.000168
MEF2C	0.072	2.39E-09	-0.01	0.119	0.115	5.33E-19	-0.06	0.000389
MEIS3	0.083	3.64E-07	0.094	3.79E-25	0.12	2.22E-19	0.003	0.545
MNDA	0.04	0.000217	0.03	0.0032	0.106	1.82E-16	0.022	6.94E-05
MRPL12	-0.053	1.73E-10	0.022	0.00148	-0.043	6.94E-07	-0.026	7.9E-08
MSC	0.089	1.2E-14	0.104	1.16E-23	0.097	3.11E-19	0.012	0.0445
MSRB2	0.011	0.106	0.001	0.735	-0.014	0.0768	-0.016	1.02E-05
MXD3	-0.027	0.00842	0.029	0.000383	-0.037	5.24E-05	-0.007	0.673
MYOCD	0	1	-0.057	4.77E-07	-0.009	0.376	0.098	2.1E-06
MZF1	-0.103	1.37E-09	-0.062	9.99E-18	-0.288	2.46E-16	-0.003	0.771
NCF1	-0.015	0.0422	0.042	1.44E-07	0.079	5.67E-13	0.001	0.423
NFAM1	0.063	8.73E-07	0.047	4.09E-06	0.087	1.41E-18	0.006	0.102
NLRP3	0.051	7.87E-09	0.024	0.00164	0.11	5.33E-19	-0.015	0.48
NOTCH3	0.038	0.0139	0.04	3.55E-05	-0.028	0.0107	0.023	3.79E-08
NOTCH4	-0.013	0.388	0.042	6.73E-08	0.064	9.67E-14	-0.007	0.424
OSM	0.055	1.83E-05	0.033	0.00325	0.109	1.13E-15	0.031	1.32E-06
PABPC1L	0	1	-0.055	1.14E-19	-0.032	2.17E-07	0	1
PAXBP1	-0.043	0.000456	-0.065	1.95E-18	-0.078	1.04E-18	-0.009	0.02
PDE2A	0.067	5.24E-09	0.055	6.46E-08	0.058	5.21E-07	0.027	6.11E-10
PIDD1	-0.083	3.59E-08	-0.006	0.638	-0.083	4.08E-15	-0.045	8.93E-10
PLEK	0.034	0.00779	0.072	2.65E-11	0.103	2.49E-16	0.005	0.415
PLK1	-0.035	0.00126	0.062	1.33E-14	-0.027	0.00434	0.002	0.961
PNN	0.017	0.161	-0.013	0.212	-0.026	2.5E-05	-0.032	2.33E-08
POU2AF1	-0.009	0.6	0.01	0.361	0.057	0.000113	0.002	0.781

PRRX1	0.063	5.25E-07	0.088	2.31E-18	0.151	7.58E-22	0.052	9.81E-10
PSMB9	-0.042	5.9E-05	0.061	3.78E-14	0.083	3.96E-10	-0.022	8.36E-07
PTGES2	-0.073	3.37E-13	0.02	0.00461	-0.019	0.028	-0.006	0.191
PYHIN1	0.004	0.897	0.055	2.23E-08	0.109	4.41E-16	-0.009	0.0312
RBM39	-0.027	0.024	-0.071	3.93E-21	-0.033	1.17E-07	-0.002	0.937
RECQL5	-0.086	3.34E-09	-0.033	1.99E-06	-0.047	2.41E-07	-0.016	0.00156
RHOH	0.002	0.937	0.053	5.16E-07	0.075	1.05E-10	0.007	0.127
S100A9	-0.045	1.35E-08	0.033	3.02E-06	0.034	0.00108	0.026	2.94E-07
S1PR1	0.058	1.82E-08	0.001	0.912	0.107	1.46E-16	0.043	2.99E-10
SAP18	-0.078	1.91E-08	-0.026	4.41E-05	0.027	0.0353	-0.017	0.00112
SARNP	-0.022	0.00803	0.003	0.759	-0.059	4.05E-18	-0.017	0.00692
SFRP4	0.12	5.39E-18	0.023	0.0211	0.113	1.62E-17	-0.004	0.638
SLC11A1	0.074	1.02E-10	0.034	2.28E-06	0.065	5.54E-09	0.011	0.0889
SLIRP	-0.039	1.88E-06	0.024	0.000684	-0.052	2.7E-16	-0.019	0.00271
SMO	0.016	0.0104	0.087	3.19E-25	-0.044	6.22E-05	0.013	0.000743
SOX17	0.125	2.08E-11	0.069	1.99E-07	-0.108	1.52E-18	0.059	3.74E-08
SOX18	-0.007	0.583	0.017	0.0818	0.072	1.97E-10	-0.007	0.185
SPI1	0.071	3.64E-09	0.027	0.00563	0.081	2.76E-12	0.014	0.0443
STK36	-0.12	4.55E-09	-0.046	2.93E-13	-0.106	2.64E-20	-0.011	0.00298
SULF1	0.046	1.94E-07	0.099	2.55E-24	0.075	3.22E-05	-0.036	9.6E-05
TAF1C	-0.021	0.224	-0.042	5.53E-10	-0.058	3.78E-11	-0.012	0.00756
TAF1D	0.003	0.6	-0.034	2.02E-05	-0.018	0.00664	0.003	0.688
TCEA3	-0.078	5.54E-11	-0.071	7.8E-21	0.015	0.606	0.004	0.0647
TFEC	0.058	4.33E-05	0.03	0.00106	0.069	4.22E-13	-0.008	0.0701
TGFB1	0	1	0.048	2.79E-05	0.133	3.88E-16	0.033	0.000199
TGFB1I1	0.065	6.17E-11	0.066	1.15E-12	0.088	4.07E-19	0.011	0.0748
TGFB2	0.103	2.24E-11	0.008	0.735	0.031	0.000122	0.025	0.105
TLR2	0.043	0.161	0.005	0.931	0.083	3.85E-14	0.014	0.269

TLR4	0.088	1.13E-11	0.018	0.15	0.108	8.07E-19	-0.023	0.761
TMIGD3	0.05	0.00944	0	1	0.079	7.2E-11	0.008	0.0504
TNFAIP3	0.101	1.9E-15	0.067	8.33E-13	0.074	2.97E-10	-0.05	1.36E-11
TNFSF8	0.006	1	0.029	0.00361	0.054	2.76E-13	0.011	0.0514
TOP2A	-0.028	0.00944	0.062	3.12E-14	-0.008	0.468	-0.016	0.00122
TRIM52	-0.013	0.53	-0.081	3.51E-21	-0.03	0.000433	-0.027	8.39E-08
TSSK4	0	1	-0.044	8.72E-15	-0.05	9.07E-15	0.005	0.397
VGLL3	0.104	2.32E-09	0.074	9.98E-15	0.097	4.08E-15	0.022	9E-05
ZC3H8	-0.051	8.27E-07	-0.041	1.5E-05	-0.073	2.3E-10	-0.017	1.34E-05
ZEB1	0.068	1.72E-08	0.021	0.0933	0.1	5.33E-19	0.055	4.32E-08
ZEB2	0.096	5.11E-14	0.048	5.65E-07	0.137	2.24E-20	0.008	0.00588
ZNF133	0.012	0.346	-0.007	0.704	-0.128	4.35E-15	-0.006	0.638
ZNF169	0	1	-0.074	3.38E-21	-0.054	7.39E-13	-0.009	0.638
ZNF19	0	1	-0.087	1.69E-20	-0.073	3.32E-14	-0.001	0.981
ZNF23	0.079	9.86E-07	-0.055	8.39E-14	-0.046	0.000511	-0.018	0.00109
ZNF276	-0.091	1.2E-13	-0.062	1.38E-19	-0.02	0.000928	-0.011	0.125
ZNF337	-0.04	3.75E-07	-0.039	9.7E-07	-0.049	8.23E-12	-0.017	2.1E-06
ZNF354B	-0.004	0.585	-0.082	1.91E-21	-0.052	8.36E-07	-0.014	0.00271
ZNF469	0.087	5.73E-15	0.048	1.42E-06	0.106	1.04E-18	0.09	1.06E-08
ZNF688	-0.063	4.18E-06	-0.041	1.89E-06	-0.042	9.77E-09	-0.057	2.23E-07
ZNF692	-0.033	0.000344	-0.052	5.69E-13	-0.069	4.72E-15	-0.022	3.74E-08
ZNF785	0	1	-0.048	2.69E-10	-0.087	1.31E-15	0	1
ZNF789	-0.03	0.000719	-0.024	0.000455	-0.063	3.67E-14	-0.051	1.48E-10
ZNF862	0	1	-0.056	4.93E-15	-0.015	0.166	0	0.952
ZRANB2	-0.029	0.00272	-0.115	9.43E-27	-0.091	1.16E-17	-0.003	0.826
**Diff. Activated**		**116**		**129**		**138**		**84**
**Missing Mrs**		**16**		**3**		**3**		**8**

Table S7: Differentially activated MRs (out of 156 MRs) in the PRECOG cancer datasets for HNSC, LUAD, OV and SKCM cancer types.
